# Landscape genomics reveals adaptive genetic differentiation driven by multiple environmental variables in naked barley on the Qinghai-Tibetan Plateau

**DOI:** 10.1038/s41437-023-00647-0

**Published:** 2023-11-08

**Authors:** Tongrui Chen, Jinqing Xu, Lei Wang, Handong Wang, En You, Chao Deng, Haiyan Bian, Yuhu Shen

**Affiliations:** 1 Key Laboratory of Adaptation and Evolution of Plateau Biota, Laboratory for Research and Utilization of Qinghai Tibetan Plateau Germplasm Resources, Qinghai Provincial Key Laboratory of Crop Molecular Breeding, Northwest Institute of Plateau Biology, Chinese Academy of Sciences, Xining, 810000 China; 2 University of Chinese Academy of Sciences, Beijing, 100049 China; 3 Innovation Academy for Seed Design, Chinese Academy of Sciences, Xining, 810000 China

**Keywords:** Plant evolution, Structural variation

## Abstract

Understanding the local adaptation of crops has long been a concern of evolutionary biologists and molecular ecologists. Identifying the adaptive genetic variability in the genome is crucial not only to provide insights into the genetic mechanism of local adaptation but also to explore the adaptation potential of crops. This study aimed to identify the climatic drivers of naked barley landraces and putative adaptive loci driving local adaptation on the Qinghai-Tibetan Plateau (QTP). To this end, a total of 157 diverse naked barley accessions were genotyped using the genotyping-by-sequencing approach, which yielded 3123 high-quality SNPs for population structure analysis and partial redundancy analysis, and 37,636 SNPs for outlier analysis. The population structure analysis indicated that naked barley landraces could be divided into four groups. We found that the genomic diversity of naked barley landraces could be partly traced back to the geographical and environmental diversity of the landscape. In total, 136 signatures associated with temperature, precipitation, and ultraviolet radiation were identified, of which 13 had pleiotropic effects. We mapped 447 genes, including a known gene *HvSs1*. Some genes involved in cold stress and regulation of flowering time were detected near eight signatures. Taken together, these results highlight the existence of putative adaptive loci in naked barley on QTP and thus improve our current understanding of the genetic basis of local adaptation.

## Introduction

Natural and artificial selection have varying degrees of influence on plant genomes. The effects on genetic polymorphisms are reflected in adaptive differences among different crop cultivars (Zhao et al. 2013; Li et al. 2020a, 2020b). Identifying the genomic and geographic extent of adaptation to better understand the genetic regulation of crop adaptation to the environment is one of the enduring goals of evolutionary genetics. Natural selection is one of the key processes by which plants adapt to the environment, and it directly affects the adaptive traits and associated regions in the genome (Zhao et al. 2013). In this process, alleles that provide an adaptive advantage to the specific environment become prevalent. Identification of these adaptive genes is critical for understanding plant adaptive evolution and crop improvement.

Landscape genomics offers an approach to understanding local adaptation. It is an emerging research field that combines landscape factors and genomics to identify the environmental factors that shape adaptive genetic variation and the gene variants that drive local adaptation (Allendorf et al. 2010; Schoville et al. 2012; Rellstab et al. 2015). Its development has been facilitated by next-generation sequencing (NGS) and improvements in datasets describing environmental factors. Of late, numerous analytical methods for environmental association studies have been developed, and outlier loci based on population genetic differentiation have been found based on environmental associations. These provide not only a basis for understanding the impact of geographic and ecological factors on spatial genetic patterns and adaptation but also a foundation for gaining insights into the genetic mechanisms of local adaptation (Li et al. 2018; Zhang et al. 2019a; Zhao et al. 2020). For instance, Abebe et al. (2015) reported several outlier loci in Ethiopian barley associated with temperature and altitude. Lasky et al. (2015) used genome−environmental associations in sorghum landraces to predict adaptive traits. Their results suggested that genomic signatures of environmental adaptation might be useful for crop improvement, enhancing germplasm identification and marker-assisted selection. Russell et al. (2016) identified signatures highly associated with environmental factors, and these signatures clustered in different genomic regions. This study provided insights into the environmental adaptation of geographically diverse barley. Six previously reported adaptive loci were identified using *F*st outlier and mixed-model association analysis, and 282 new barley genes were also identified by this method (Lei et al. 2019). Chang et al. (2022) identified significant correlations with environmental variables and strong genetic differentiation in the pericentromeric regions on chromosomes 3H, 4H, and 5H of southern Levant wild barley. On the other hand, landscape genomics could be useful to evaluate the adaptability potential of crops to current and future climate and to identify the areas where crops would be at great risk under future climate conditions. Thus, the breeders could evaluate the agro-biodiversity potential of local varieties to mitigate the impact of climate change and propose suggestions for breeding efforts targeting crops' local adaptation (Rhoné et al. 2020; Caproni et al. 2023). In addition, landscape genomics could provide a basis for understanding the evolutionary history of species (Zhao et al. 2020). Gutaker et al. (2020) used population-genomic analyses to examine the environmental factors associated with the geographic distribution of rice diversity, thus reconstructing the ancient distribution of rice in Asia and the history of rice spreading outward from its origin center. These studies have identified markers and genes potentially involved in local adaptation and demonstrated the utility of landscape genomics in detecting and understanding the adaptive biology of plants.

The Qinghai-Tibetan Plateau (QTP) is famous for being the highest and largest plateau in the world, with an average altitude exceeding 4000 m. The high altitude results in strong solar radiation, low temperature, a large temperature difference between day and night, dry climate, low annual precipitation, and an obvious regional microclimate. The harsh habitats force plants living on QTP to adapt to extreme conditions. Therefore, QTP provides an ideal ecological environment for dissecting the genetic basis of plant ecological adaptation. Barley (*Hordeum vulgare* L.) is one of the founding crops of Old-World agriculture (Badr et al. 2000). Naked barley is the main crop on QTP and has been used as a major staple food by Tibetans for generations (Dai et al. 2012). It was derived from eastern domesticated barley, which most likely passed through northern Pakistan, India, and Nepal between 4500 and 3500 years ago and had been cultivated on QTP for at least 3500 years (Zeng et al. 2018). Naked barley is generally sown in April and harvested from August to September, and grows once a year on QTP. The growth period involves a summer with high levels of UV-B radiation (approximately 65 kJ/m^2^ in summer) (Norsang et al. 2009) and low temperatures (average yearly temperature 7.6 °C) (Zhang et al. 2015). Therefore, naked barley has been domesticated under natural and artificial selection pressures that are quite different from those for cultivated barley from other regions (Zeng et al. 2018). Tibetans usually sow their own harvested grain as seeds from year to year, thus establishing farmers’ varieties (landraces) adapted to different ecological environments across QTP.

Recently, landscape genomics has developed vigorously and has been popularized and applied to barley (Abebe et al. 2015; Russell et al. 2016; Lei et al. 2019; Chang et al. 2022; Caproni et al. 2023), rice (Gutaker et al. 2020), sorghum (Lasky et al. 2015), and pearl millet (Rhoné et al. 2020), but the cultivation of naked barley on QTP has been still relatively scarce. In this study, our specific aim was to use a landscape genomics approach to detect the local adaptation characteristics of naked barley populations on QTP. Here, we describe the population structure of naked barley landraces from QTP. Then, we characterize the relative contributions of environmental variables and geographic distance using partial redundancy analysis (pRDA). Finally, we report on several putative adaptive loci associated with environmental variables and identify candidates for local adaptation-related genes. We assumed that under the harsh environment of high altitude, cold, and strong ultraviolet radiation on QTP, the adaptation of naked barley might be jointly regulated by multiple genes. Detection of these signatures of local adaptation will help in understanding the adaptation mechanism of naked barley and other major crops on QTP.

## Materials and Methods

### Sample collection and genotyping

**Fig. 1:**
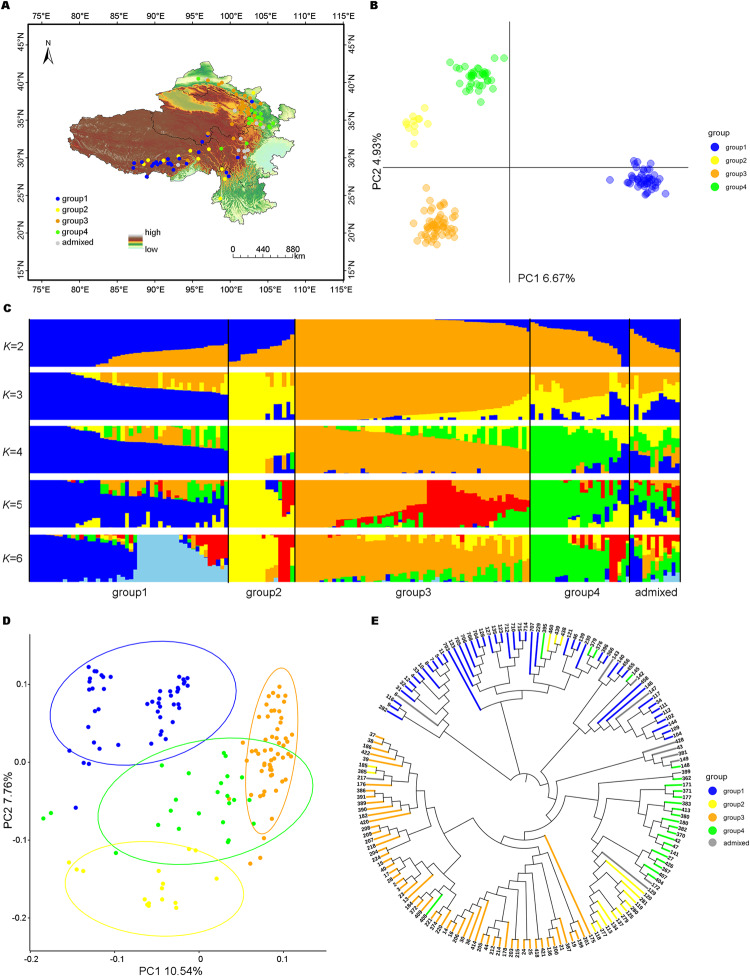
Inference of population genetic structure. **A** Distribution of 157 naked barley landraces. Blue, yellow, orange, and green dots correspond to Group 1, Group 2, Group 3, and Group 4, respectively. **B** The first two axes of the principal component analysis on SNP markers. Individual genotypes were colored based on the cluster allocation of the discriminant analysis of principal components (DAPC). Colors are explained in the legend to the right. The axes denote the relative proportions of explained genetic variance. **C** Individual ancestry coefficients of 157 naked barley landraces estimated using Admixture with *K* = 2–6. With *K* = 4, blue, yellow, orange, and green bars correspond to Group 1, Group 2, Group 3, and Group 4, respectively. **D** Principal component analysis. **E** Neighbor-joining tree.

A total of 157 naked barley landraces originating from QTP were used as the study samples. The accessions originated from the following QTP areas: Qinghai (51), Tibet (38), Gansu (46), Sichuan (15), and Yunnan (7) (Fig. 1A and Table S1). In general, the 157 naked barley accessions provided a good representation of the landraces in QTP geographically.

DNA was extracted from young leaves using the CTAB protocol (Doyle and Doyle 1990). Genotyping-by-sequencing (GBS) was performed following the procedure reported by Elshire et al. (2011). Briefly, genomic DNA was digested with the restriction enzyme *ApeK* I (G|CWCG), barcoded libraries were prepared to track each accession, and the DNA sequence corresponding to the region flanking the *ApeK* I site was obtained using the Illumina HiSeq 2000 platform. The raw sequence data were filtered by SOAPnuke (http://soap.genomics.org. cn/). Then, clean sequences were mapped to the *Hordeum vulgare* Hv IBSC PGSB v2 reference genome (Mascher et al. 2017) using BWA (Li and Durbin 2009), and raw SNPs were generated by GATK (McKenna et al. 2010). The SNPs generated by GATK were further filtered using VCFtools ver. 0.1.13 (Danecek et al. 2011). In total, 118,183 polymorphic sites for each accession were discovered. The data used for the population structure analysis and pRDA were filtered as follows: missing values ≤ 0.2, heterozygosity rate (Het. Rate) ≤ 0.2, and minor allele frequency (MAF) ≥ 0.1. Then, the selected 28,022 SNPs were pruned using PLINK ver. 1.9 (Purcell et al. 2007) to remove SNPs in linkage-disequilibrium (LD) with an *r*^*2*^ threshold of 0.5, a window size of 150, and a step size of 5, which eventually resulted in 3123 SNPs. For outlier analysis, SNPs with MAF ≤ 0.05, heterozygosity rate (Het. Rate) ≥ 0.2, and missing data rate ≥ 0.2 were removed from 118,183 SNPs. After filtering, 37,636 high-quality SNPs were retained, and they were annotated for functional effects based on the reference genome using SnpEff (Cingolani et al. 2012).

### Extraction of environmental variables

Environmental variables can be divided into two parts: geographic factors and climate variables. The geographic factors included latitude, longitude, and altitude. Data for the climate variables (temperature, precipitation, and solar radiation) during the growth period were downloaded from WorldClim (http://www.diva-gis.org/climate) for 1970–2000 at 30-s resolution, and 30 variables (Tables S2 and S3) were extracted using DIVA-GIS ver. 7.5 (Hijmans et al. 2001).

### Population structure analysis

A phylogenetic tree was constructed based on the distance matrix calculated by MEGA ver. 11 (https://megasoftware.net/), and the output was visualized using iTol (https://itol.embl.de/) (Letunic and Bork 2016). Genetic groupings of samples were identified with discriminant analysis of principal components (DAPC) using the R package “*adegenet*” (Jombart et al. 2010). Principal component analysis (PCA) was performed with EIGENSOFT ver. 7.2.1 (Patterson et al. 2006). Analysis of the hierarchical population structure was done using ADMIXTURE ver. 1.3.0 (Alexander et al. 2009) for *K* values from 2 to 10; for each value of *K*, 5 independent runs were performed. The *CV* error values were calculated for *K* values from 2 to 10.

To analyze the genetic differentiation, we calculated *F*st and AMOVA between genetic clusters defined by population structure analysis. *F*st values were calculated using VCFtools ver. 0.1.13 and AMOVA was undertaken using Arlequin ver. 3.5 (Excoffier and Lischer 2010).

### Partial redundancy analysis

Partial redundancy analysis was performed using the function *rda* of the classic R package “*vegan*” (Oksanen et al. 2022). We used the set of LD-pruned SNPs (*r*^*2*^ = 0.5) as response variables, while geography, climate, and genetic structure were treated as explanatory variables. First, the missing values of the SNP dataset were obtained by simple imputation, and then, the 3123 SNPs were converted to 0, 1, and 2 forms, which represented homozygosity for the most frequent allele, and heterozygosity and homozygosity for the least frequent allele (Capblancq and Forester 2021). Geography was represented by the latitude and longitude of the naked barley landraces; the climate was represented by noncollinear climate variables at each material sampling point; genetic structure was represented by the first three axes of the genetic PCA marked with the same set of LD-pruned markers. To examine how much of the genetic variation in naked barley landraces could be explained by geography, climate, genetic structure, and the combination of the three, we used pRDA. The variance components of RDA were partitioned by four different models. The first model used geography, climate, and genetic structure as explanatory variables; the second model used genetic structure as the explanatory variable, and climate and geography as covariates; the third model used geography as the explanatory variable and was conditioned on climate and genetic structure; the fourth model used climate as the explanatory variable and controlled the remaining components (Capblancq and Forester 2021). We then used pRDA to look for genotype−environment associations, by employing a pure neutral population structure model (controlling genetic structure) and the six noncollinear climate indicators as explanatory variables. The adaptive loci were determined based on their position along the Mahalanobis distance distribution calculated for each marker and the center of the RDA space using the first four axes; the distances were then corrected for the inflation factor to derive *p*-values using a chi-squared distribution with two degrees of freedom (Capblancq et al. 2018). The Bonferroni threshold with a nominal *p-*value of 10% was used to identify outliers. The analysis was carried out using the R package “*vegan*” (Oksanen et al. 2022) and outliers were detected with the R function *rdadapt*.

### Genome−environment association

To detect the associations between the environment and SNPs, we used two conceptually different methods: latent factor mixed model (LFMM) (Caye et al. 2019) and environment genome-wide association study (EnvGWAS) (Li et al. 2019).

LFMM is a univariate genome−environment association method. This analysis was performed using the R package “*lfmm*” (Caye et al. 2019). Before performing the analysis, the 37,636 high-quality SNPs were converted to 0, 1, and 2 forms; the missing values of the SNP dataset were assigned by simple imputation as in RDA. The 30 original environmental variables were used for association analysis. We chose the first two principal components generated for genetic markers as latent factors to estimate the population structure effect. The analysis was run five times to increase the credibility of the association. To detect the differential adaptive loci, we used Storey’s *q*-values method (Storey et al. 2023) and the false discovery rate (FDR = 0.05) as the significance level. In addition, we determined the fitting degree based on QQ plots.

EnvGWAS represents the association between SNP alleles and the original environment. In this analysis, we used a Fixed and random model Circulating Probability Unification (FarmCPU) implemented by a memory-efficient, visualization-enhanced, and parallel accelerated tool (MVP, https://github.com/xiaolei-lab/rMVP). FarmCPU is a method aimed at overcoming the limitation of the general linear model (GLM) and mixed linear model (MLM) in dealing with population structure, so as to generate a higher statistical capacity (Liu et al. 2016). The EnvGWAS was run on 37,636 high-quality SNP markers and the missing values of the SNP data were imputations by the native program. The set of SNP markers pruned by LD at a threshold of *r*^*2*^ = 0.5 were used to calculate the kinship and genetic PCs. The genetic structure in the panel was corrected using 2−4 genetic PCs. The 30 original environmental traits were used as response variables. For each trait, the most suitable number of PCs was obtained based on the fitting degree of the QQ plots. When the SNP markers surpassed the stringent threshold of a Bonferroni correction with α = 0.05 and the less stringent threshold of FDR = 5%, these markers were identified as significantly associated loci. FDR was computed with the R package “*qvalue*” (Storey et al. 2023). For each of these 30 test traits, we estimated the proportion of phenotypic variance explained by each significant SNP after FDR correction (Teslovich et al. 2010).

### Detection of candidate genes

Linkage disequilibrium (LD) was evaluated as the pairwise *r*^*2*^ of 37,636 SNPs using PopLDdecay ver. 3.40 (Zhang et al. 2019b) with a maximum distance of 300 kb. The search for positional candidate genes was performed based on the chromosome-specific LD decay distance. The candidate interval corresponding to each significance locus was defined within the chromosome-specific LD decay distance flanking each SNP marker that was associated with the environmental traits. The genes located in the candidate intervals were considered candidate genes. We used the BLASTp function of the protein sequence analysis to find genes homologous to the candidate genes and used the Pfam database (https://pfam.xfam.org/) to find the function of the protein domain. Enrichment of these genes could be performed using the “GO Enrichment” option implemented in TBtools (Chen et al. 2020).

We used a sliding window approach (window size = 20,000 bp, step size = 10,000 bp) to describe the pattern of variation and over-divergence across the genome (Cortés et al. 2018a, 2018b). We used Tassel ver. 5 (Bradbury et al. 2007) to compute per-window averages of SNP density, nucleotide diversity (pi) (Nei 1987), Tajima’s D (Tajima 1989), and Watterson’s theta estimator (θ) (Watterson 1975). Then we calculated the whole-genome SNP density, pi, Tajima’s D, and θ differences between candidate intervals and no-candidate intervals in the genome window.

## Results

### Genotyping-by-sequence

Approximately 1.31 TB of clean reads were generated by GBS, with an average of 18,544,144 reads per sample aligned to the barley reference genome (Mascher et al. 2017), and the genomic coverage for each sample ranged from 2.31% to 5.12% (Table S4). The entire set of original sequence data has been deposited in the Genome Sequence Archive (https://ngdc.cncb.ac.cn/gsa/) in the BIG Data Center at the Beijing Institute of Genomics (BIG), Chinese Academy of Sciences, under accession number CRA007788. For analysis of population genetic structure and pRDA, we selected 3123 SNPs with MAF ≥ 0.1, Het. Rate ≤ 0.2, missing values ≤ 0.2, and overall missingness of 6.42%; this dataset was LD-pruned with PLINK using an *r*^*2*^ threshold of 0.5. In this dataset, 707 (22.64%) SNPs were located in the exon region (Table S5). For analysis of genome−environment association, after filtering for MAF ≥ 0.05, Het. Rate ≤ 0.2, and missing data ≤ 0.2, 37,636 high-quality SNPs with an overall missingness of 6.04% were retained. Most SNPs (49.97%) were presented in intergenic regions, and 17.38% of the SNPs were presented in coding regions. There were 3138 (0.83%) missense variants, 8 (0.02%) start-loss mutations, 55 (0.15%) stop-gain mutations, and 7 (0.02%) stop-loss mutations capable of leading to significant functional mutations (Table S6).

### Analysis of population genetic structure

To provide an unbiased representation of the existing diversity, a smaller set of 3123 SNP markers was derived by LD pruning. The analysis of population structure among 157 naked barley landraces with DAPC (Jombart et al. 2010) identified four groups (Fig. S1A). The first two principal components (PCs) axes explained 6.67% and 4.93% of the variation, respectively (Fig. 1B). At the same time, the *CV* error values showed a turning point at *K* = 4 (Fig. S1B). We plotted a hierarchical population structure with *K* = 2–6 and identified 12 mixed individuals at *K* = 4 (Fig. 1C), which were mostly located at the border of the provinces (Fig. 1A). Then, we combined the results of DAPC and population structure analysis to ultimately divide 145 naked barley landraces into four groups. The four groups included 48, 16, 57, and 24 individuals, respectively. With *K* = 4, 64 of 145 (44.1%) individuals had a higher ancestry coefficient of more than 0.8. There were 17 (35.4%), 9 (56.3%), 28 (49.1%), and 10 (41.7%) individuals with membership coefficients of Q ≥ 0.8 in Group 1, Group 2, Group 3, and Group 4, respectively. The first two PCs represented the groups corresponding to the previously determined results. On the first PC axis, Group 3 was separated from the other three groups. On the second PC axis, Group 1, Group 4, and Group 2 could be distinguished from each other. The confidence interval was 0.95 and the two PC axes explained 10.54% and 7.76% of the variation, respectively (Fig. 1D). The phylogenetic tree displayed the same hierarchical structure as the population structure analysis (Fig. 1E). In terms of the geographical distribution of each group of naked barley landraces, we found that the geographical sources of accessions in Groups 1 and 2 were similar, both including the regions of one river and two streams. The difference was that the extension area of Group 1 covered the border of Qinghai, Tibet, Sichuan, and Yunnan, while the extension area of Group 2 was mainly located within Yunnan. The individuals in Group 3 were mainly from Hehuang Valley in Qinghai and Hexi Corridor of Gansu. The landraces in Group 4 mainly correspond to southern Gansu. In addition, the results showed that the first and second groups were distributed in low latitudes, while the third and fourth groups were distributed in high latitudes (Fig. 1A), which indicated that the distribution of the naked barley group had a certain correlation with the latitude of its origin. From the perspective of geographical distribution, Groups 1 and 2 could be categorized as the southern group, while Groups 3 and 4 could be categorized as the northern group (Fig. 1A).

To quantify the extent of genetic differentiation among the four groups, we calculated *F*st and performed AMOVA. Group 2 was most strongly isolated from the other three groups, and there was a moderate degree of genetic differentiation among Group 1, Group 3, and Group 4 (Table S7). The AMOVA results showed that genetic variation within the populations accounted for 82.81% of the total variation, indicating a relatively rich genetic diversity within the populations (Table S8).

### Genetic variation explained by geographic and climate

**Fig. 2:**
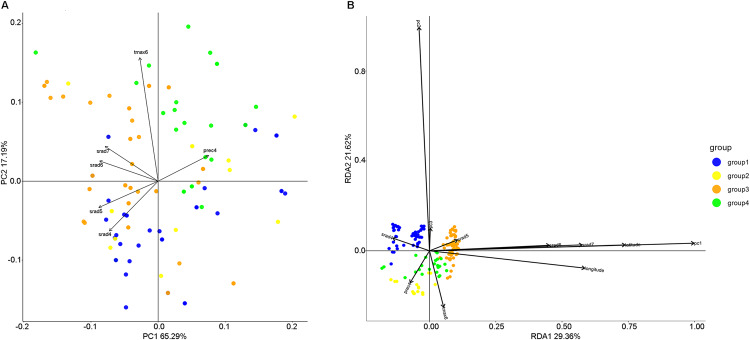
Results of principal component analysis of climate variables and RDA biplot. **A** Principal component analysis of the climatic diversity of 145 georeferenced barley landraces. The axes denote the proportion of explained climatic variance; the dots’ colors represent the groups corresponding to the results of population structure analysis, and the vectors represent the scale, diversity, and direction of drivers of differentiation. **B** RDA showed that the six nonredundant climate variables were correlated with the observed population structure and might have had selective impacts in the past. The biplot depicted the eigenvalues and length of eigenvectors for the RDA. Blue, yellow, orange, and green dots correspond to Group 1, Group 2, Group 3, and Group 4, respectively.

The six nonredundant climate variables were identified using *ordiR2step* function, srad4 (solar radiation in April), srad5 (solar radiation in May), srad6 (solar radiation in June), and srad7 (solar radiation in July) related to solar radiation, while prec4 (precipitation in April) and tmax6 (maximal temperature in June) described the precipitation and temperature patterns, respectively (Table S9). The climate variables showed an extensive difference throughout the entire sampling area, which can be observed by PCA. The first climatic PC was positively correlated with prec4 and negatively correlated with srad5, srad6, and srad7, accounting for 65.29% of the climatic variance on the collecting sites of naked barley landraces. The second climatic PC was positively associated with tmax6 and explained 17.19% of the climatic variance (Figs. 2A and S2A). Climatic PC variables could detect the groups identified by population structure analysis, and the distribution pattern was related to ultraviolet radiation, temperature, and precipitation (Fig. 2A). The southern groups (Groups 1 and 2) were cultivated in strong ultraviolet radiation and warmer climates, while the northern groups (Groups 3 and 4) were cultivated in low ultraviolet radiation, and colder and dry climates. Group 2 was cultivated in the wettest, warmest, and highest ultraviolet radiation conditions compared with the other groups, while Group 4 was cultivated in the coldest and lowest ultraviolet radiation climates (Table S10).

Four different partial redundancy models were used to validate the genomic variations accounted by genetic structure, geographic distance, and climate variables. Model 1 indicated that the combination of genetic structure, climate, and geographic distance explained 24.42% of the variation (Table S11). In this model, the biplot showed a population structure consistent with the four groups identified by population structure analysis, with the ratios of axes 1 and 2 being 29.36% and 21.62%, respectively (Fig. 2B). Model 2 showed that the influence of genetic structure accounted for almost half (45.58%) of the explained variation (Table S11). Model 3 indicated that the geographical coordinate was only responsible for 6.86% of the explainable variance, while the effect of climate on the control of neutral genetic structure and geography was significant and accounted for up to 21.73% of the genotypic variation (Model 4), which suggested that climate variables might play a more significant role in explaining genomic variation (Table S11). When controlling for genetic structure, the first RDA axis was strongly correlated with the variables tmax6 and srad7 (Fig. S2B and Table S12). In addition, we found that 25.83% of the explainable variance was not directly related to genetic structure, geography, and climate variables (Table S11).

### Detecting putative adaptive loci

We regressed the set of LD-pruned SNP markers against the six nonredundant climate variables using pRDA with control on the genetic structure. Finally, we found only one candidate SNP. The adaptive locus is located at 492,394,013 bp on chromosome 5H and was identified in 5’-UTR (Table S13 and Fig. S3). In LFMM, a total of 92 candidate SNPs were detected and associated with five climate variables. Of the 92 loci, the largest number of loci (21) was located on chromosome 7H, while the lowest number (8) was located on chromosomes 4H and 5H. In addition, the largest number of loci (42) was associated with prec5, and only one locus was associated with tmax9 (Table S13 and Fig. S4). In total, five loci had pleiotropic effects and all of them were only associated with temperature or precipitation variables. Among them, four loci were associated with precipitation variables in different months and only one locus at 183,999,899 bp on chromosome 3H was associated with temperature variables, tavg9 and tmax9 (Table S13). The significant SNPs identified by LFMM showed a high ratio of loci (55.43%) located in the intergenic regions, and 10 (10.87%) were located in the exon (Table S14). In the EnvGWAS, the associations were identified using the first 3 PCs, and a total of 45 signatures were distributed across the naked barley genome that was associated with 11 climate variables (Table S13 and Fig. S5). Of the 45 loci, 8 loci had pleiotropic effects, of which 7 loci were only associated with temperature, precipitation, or ultraviolet radiation in different months, while only 1 locus could be co-associated with precipitation and ultraviolet radiation. For example, a locus with pleiotropic effects located at 650,367,670 bp on chromosome 5H was only associated with temperature variables, while the locus at 452,485,038 bp on chromosome 5H was related to the five climate variables, prec6, prec7, prec9, srad7, and srad8 (Table S13). Of the 45 significant SNPs, 37.78% were identified in intergenic regions and 26.67% were located in genic regions (Table S14). It is worth mentioning that one locus at 623,414,235 bp on chromosome 7H was identified in a stop-gained mutation (Table S13).

In general, a total of 136 loci were identified by the 3 methods, of which 13 loci had pleiotropic effects and were associated with more than 2 climate variables (Table S13). The largest number of SNPs (32) was located on chromosome 7H and the lowest number of loci (9) was located on chromosome 4H. The largest number of loci (48) was associated with prec5 and only 1 locus was associated with tmax9 (Table S15). Two common SNPs were identified by LFMM and EnvGWAS: one locus at 720,353,705 bp on chromosome 2H was associated with prec5 and was located in the upstream gene region; the other locus at 628,314,429 bp on chromosome 4H was associated with srad4, prec4, and srad9, and was identified in missense mutation (Table S13). The annotation conducted on the 136 significant SNPs showed that a high ratio of loci (50%) was identified in intergenic regions and 15.44% were located in genic regions. In addition, a total of 21 (15.44%) and 8 (5.88%) loci were located in the upstream and downstream gene regions, respectively, and 5 (3.68%) loci were located in the intron regions (Table S14).

### Identification of candidate genes

**Fig. 3:**
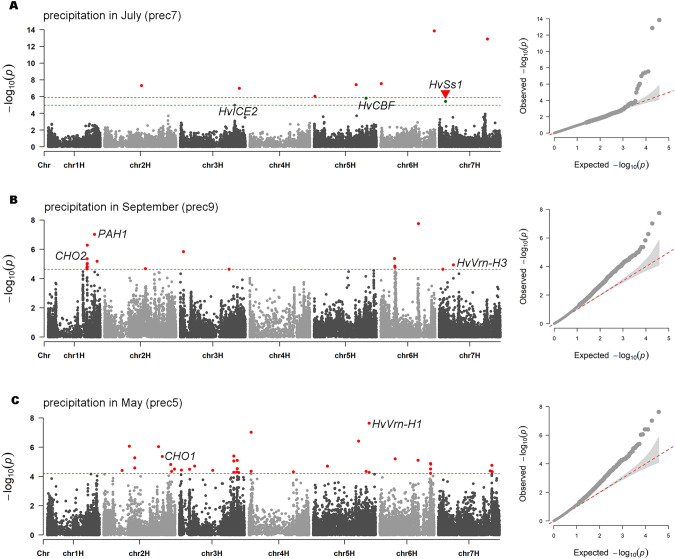
Identification of outliers. **A** The signatures identified using EnvGWAS on the 37,636 SNPs with MAF ≥ 0.05 for precipitation in July. The dark red line refers to Bonferroni correction based on α = 0.05 (− log10 (*p*) = 5.877), while the green line is based on a false discovery rate with a q-value > 0.05. The red dots indicate the significant loci. **B**, **C** The candidate SNPs identified using LFMM on the 37,636 SNP markers with MAF ≥ 0.05 for precipitation in September and May, respectively. The dark green line is based on a false discovery rate with a q-value > 0.05, specific for each trait. The red dots indicate the significant loci.

To find the genes corresponding to the signatures identified by the three methods, we calculated the LD decay distance of the seven chromosomes as a scale. The LD decay distances of the seven chromosomes ranged from 115 to 145 kb (Fig. S6A). Potential candidate genes were identified in the flanking sections of significant SNPs located on chromosomes 1H–7H, which were 246 kb, 250 kb, 278 kb, 230 kb, 290 kb, 238 kb, and 234 kb, respectively. In total, 447 known genes were mapped, and most of them were assigned to the “molecular function,” “cellular component,” and “biology process” categories in the gene ontology (GO) analysis (Fig. S6B). The locus identified by RDA was 13 kb upstream of *HORVU5Hr1G063410*, and the common locus at 628,314,429 bp on chromosome 4H was 57 kb upstream of *HORVU4Hr1G084360*; both the genes encode an MYB transcription factor, which has been reported to be involved in physiological processes such as plant growth and development (Table 1). A locus identified by EnvGWAS at 67,766,605 bp on chromosome 7H associated with prec7 was found to be located 34 kb downstream of *HvSs1* (Table 1 and Fig. 3A), a known gene induced by cold temperatures (Barrero-Sicilia et al. 2011). In addition, the two loci at 497,024,530 bp and 523,730,536 bp on chromosome 1H were both associated with prec9 and were located 1.8 Mb and 3.7 Mb downstream of the cold stress pathway-related genes *PAH1* (Han and Carman 2017) and *CHO2* (Koipally et al. 1996), respectively (Table 1 and Fig. 3B). A locus at 630,451,224 bp on chromosome 2H significantly associated with prec5 was found to be located 3.3 Mb downstream of the cold stress pathway-related gene *CHO1* (Han and Carman 2017) (Table 1 and Fig. 3C). We also found two candidate loci associated with prec7 that were close to cold stress genes. The locus (3H: 654,682,638) was located 1.3 Mb upstream of *HvICE2* (Skinner et al. 2006) and the locus (5H: 558,372,123) was located 1.3 Mb upstream of *HvCBF2A* and *HvCBF4B* (Stockinger et al. 2007) (Table 1 and Fig. 3A). Furthermore, three significant SNPs associated with precipitation variables were located near the genes that regulated the barley flowering time. Two loci at 600,493,261 bp and 600,578,658 bp on chromosome 5H were located 1.3 Mb and 1.4 Mb downstream of *HvVrn-H1* (Cockram et al. 2007), and *IDI7* (Yamaguchi et al. 2002) was located 0.4 Mb upstream of these two loci (Table 1 and Fig. 3C), which was an iron ABC transporter. The locus (7H: 38,883,869) was located 0.8 Mb upstream of *HvVrn-H3* (Comadran et al. 2012) (Table 1 and Fig. 3B).

**Table 1:** Distance between the signatures and the known function genes.

Chr.	Pos.(bp)	Method	bioclimate	Annotation	Gene	Function	Distance	Reference
chr1H	497,024,530	LFMM	prec9	missense_variant	*PAH1*	Cold tolerance	1.8 Mb	Han and Carman 2017
chr1H	523,730,536	LFMM	prec9	downstream_gene_variant	*CHO2*	Cold tolerance	3.7 Mb	Koipally et al. 1996
chr2H	630,451,224	LFMM	prec5	upstream_gene_variant	*CHO1*	Cold tolerance	3.3 Mb	Han and Carman 2017
chr4H	628,314,429	LFMM, EnvGWAS	prec4	missense_variant	*HORVU4Hr1G084360*	Myb domain protein 305	57Kb	
chr5H	492,394,013	pRDA		5_prime_UTR_variant	*HORVU5Hr1G063410*	Myb domain protein 36	13Kb	
chr5H	600,493,261	LFMM	prec4	downstream_gene_variant	*HvVrn-H1* *IDI7*	Flowering time tonoplast ABC transporter	1.3 Mb 0.4 Mb	Cockram et al. 2007 Yamaguchi et al. 2002
chr5H	600,578,658	LFMM	prec5	5_prime_UTR_variant	*HvVrn-H1* *IDI7*	Flowering time tonoplast ABC transporter	1.4 Mb 0.4 Mb	Cockram et al. 2007 Yamaguchi et al. 2002
chr7H	38,883,869	LFMM	prec9	intergenic_region	*HvVrn-H3*	Flowering time	0.8 Mb	Comadran et al. 2012
chr3H	654,682,638	EnvGWAS	prec7	splice_region_variant&intron_variant	*HvICE2*	Cold tolerance	1.3 Mb	Skinner et al. 2006
chr5H	558,372, 123	EnvGWAS	prec7	intergenic_region	*HvCBF2A* *HvCBF4B*	Cold tolerance Cold tolerance	1.3 Mb 1.3 Mb	Stockinger et al. 2007 Stockinger et al. 2007
chr7H	67,766,605	EnvGWAS	prec7	intergenic_region	*HvSs1*	Cold tolerance	34Kb	Barrero-Sicilia et al. 2011

**Fig. 4:**
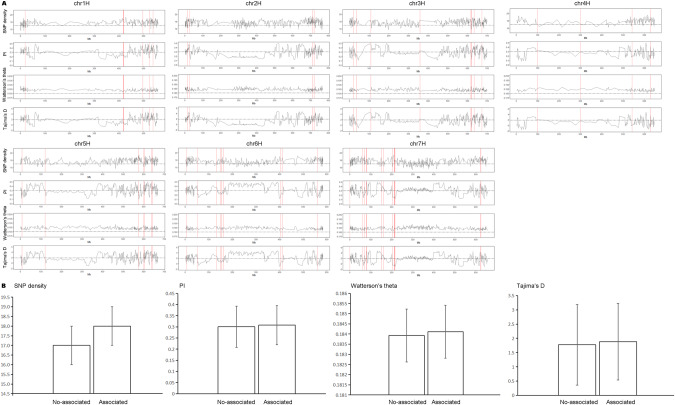
Patterns of the seven chromosomes’ diversity. **A** Patterns of the seven chromosomes’ diversity in 145 naked barley landraces based on 37,636 SNP markers. A sliding window analysis (window size = 20,000 bp, step size = 10,000 bp) was used to compute the SNP density, nucleotide diversity (pi), Watterson’s theta estimator (θ), and Tajima’s D (from top to bottom). The red lines represented the selected intervals. Gray dashed horizontal lines indicated genome averages. **B** Comparison of seven chromosomes’ diversity between associated and nonassociated intervals. The associated intervals contained at least one marker associated with climate variables. SNP density, nucleotide diversity (pi), Watterson’s theta estimator (θ), and Tajima’s D (from left to right) were calculated in a sliding window (window size = 20,000 bp, step size = 10,000 bp).

We used a sliding window analysis (window size = 20,000 bp, step size = 10,000 bp) to explore the patterns of the seven chromosomes’ diversity. The average SNP density was 17 SNPs per million base pairs, the average nucleotide diversity (pi) was 0.30 per million base pairs, the average Watterson’s theta estimator (θ) was 0.18 per million base pairs, and the average Tajima’s D was 1.87 per million base pairs (Fig. 4A). Then, these statistics were compared between candidate and noncandidate intervals. We found that the SNP density of candidate intervals was one more than that of no-candidate intervals (18 ± 1 vs. 17 ± 1); nucleotide diversity (0.308 ± 0.088 vs. 0.300 ± 0.093) and Watterson’s theta estimator (θ) scores (0.1841 ± 0.001 vs. 0.1839 ± 0.001) of candidate intervals were slightly higher than those of no-candidate intervals; and Tajima’s D scores (1.882 ± 1.342 vs. 1.776 ± 1.413) of candidate intervals were more positive than those of-candidate intervals (Fig. 4B).

## Discussion

Changing environmental conditions force organisms to become phenotypically plastic, migrate, or adapt to avoid extinction (Rellstab et al. 2015). Local adaptation is a response to selection pressure between populations and habitats, acting on genetically controlled fitness differences between individuals (Kawecki and Ebert 2004; Savolainen et al. 2013). Landscape genomics has developed rapidly in the past decade and has been proven to be an effective method for studying the adaptive evolution of species (Li et al. 2017). In the present study, we aimed to identify the climatic drivers of 157 naked barley landraces and putative adaptive loci driving local adaptation through landscape genetics and to make predictions for candidate genes.

### Correlation between the population structure of naked barley landraces and environmental variables

Understanding the role of environmental variables in forming the genetic structure of spatial populations within species is a major concern in landscape genetics (Barley et al. 2015; Leamy et al. 2016). Although such studies are popular in landscape genetics, it is still challenging to accurately determine the role of environmental factors in spatial genetic structure. The spatial genetic structure of species is the result of a combination of multiple factors, such as natural selection, genetic drift, gene flow, population demographic history, geographical or ecological distance, geographical or ecological isolation, and geologic events (Ohsawa and Ide 2008; Yang et al. 2017). In this research, population structure analysis was done using DAPC and the admixture program, supported by the principal component analysis approach and phylogenetic tree. The results showed a significant genetic divergence of naked barley landraces; the 145 landraces were significantly clustered into four genetic groups and the other 12 were mixed individuals (Fig. 1). Group 2 exhibited a high degree of genetic differentiation with the other three groups, while the other three groups exhibited a moderate degree of genetic differentiation (Table S7). At the same time, pRDA supported the division of four subgroups (Fig. 2B). The groups detected by the above results revealed a population structure that might relate to geography, which was generally divided into two major distribution areas, the south and north groups (Fig. 1A). Wang et al. (2014) also showed that the population structure of naked barley landraces on QTP was closely related to their geographic origin. Although the differentiation of the naked barley landraces on QTP might be related to the geographical origin, the interpretation of the spatial distribution of the population structure of naked barley landraces needs to consider natural selection under more environmental factors.

pRDA was performed to further verify the correlation between the population structure of naked barley landraces on QTP and environmental factors. The variance partitioning of partial RDA models showed that the variation contributed by climate variables was higher than the variation introduced by geographic variables (Table S11). Previously, Temunović et al. (2012) proposed the positive association of climate variables with genetic markers when controlling the variance caused by geographic variation, thus suggesting the importance of climate diversity in shaping genetic variation. Lasky et al. (2012) detected that the climate variation among sites of *Arabidopsis thaliana* origin explained the slightly more genomic variation than geographical distance. A similar finding was reported by Abebe et al. (2015), who found that climate factors accounted for 37.4% of the explained variation, whereas geographic position was considered to have less impact on the genetic structure of barley landraces in Ethiopia. Caproni et al. (2023) argued that climate variables had a more profound impact on barley differentiation in Ethiopia. Furthermore, the results of the forward selection process and climatic PCA demonstrated that the combination of srad4, srad5, srad6, srad7, prec4, and tmax6 was correlated with the observed population structure and might have had a selective impact in the past (Fig. 2). In the process of naked barley adaptation, temperature and precipitation are not only the two most important environmental selection pressures but also the main driving factors for the spatial distribution pattern of the population structure of barley landraces (Yahiaoui et al. 2008; Hübner et al. 2009; Jones et al. 2011).

### Identification of adaptive loci

The detection of adaptive loci is another key issue in landscape genetic research. Although adaptive loci and their environmental drivers have been identified in landscape genetic research in recent years (Di Pierroa et al. 2017; Yoder and Tiffin 2017), little attention has been given to understanding why these loci are important. Several recent studies hypothesize that environmental changes related to species' ecological habitats are the key drivers of the potential adaptive differentiation of species (Savolainen et al. 2007; Yang et al. 2017). Whether this hypothesis is universal remains to be confirmed. In this study, the association of climate variables with SNP markers using pRDA, LFMM, and EnvGWAS returned several significant loci in relation to the climate variables. We conducted a comparison between the candidate SNP markers reported in the literature and identified in our study against the barley *Hordeum vulgare* Hv IBSC PGSB v2 reference genome (Mascher et al. 2017). Notably, our study successfully reported a novel set of 136 SNP markers, which have not been previously documented. The increased number of newly identified SNP markers in our study can be attributed to several factors that warrant consideration. First, it is important to acknowledge that the field of naked barley genomics still remains relatively understudied. Second, for some reported QTLs based on SSR markers, their precise physical positions could not be found, and some reported QTLs developed using internal SNP arrays exhibited inconsistencies with the versions documented in public databases (Li et al. 2020b). Lastly, it is crucial to note that our study relied on GBS data, which inherently possess limitations associated with lower coverage. Consequently, it is possible that some previously reported SNP markers may not have been successfully identified in our analysis.

Furthermore, the intersection of SNP markers identified using three different methods was relatively less, which might be attributed to the following reasons. First, the influence of population structure needs to be considered. While LFMM and FarmCPU algorithms attempt to account for population structure during analysis, self-pollinating crops can produce false positives due to a strong genetic drift. In addition, even with corrections for population structure during computation, LFMM and EnvGWAS may still yield false positives due to the strong correlation between population structure and environmental factors (Chang et al. 2022). Second, the limitations of the dataset should be acknowledged. We selected a representative set of 157 naked barley varieties, but the sample size of this population might be insufficient. Moreover, due to the large and complex nature of the barley genome (5.5 GB), and the lower coverage of GBS data, which is not powerful enough for pinpointing the genomic targets of local adaptation (Rajendran et al. 2022; Chang et al. 2022), we may have missed strong adaptive loci among the SNP markers identified.

### Detection of candidate genes

Although there is a causal relationship between genes and phenotypes, dissecting the genetic components of a phenotype is not straightforward. With improvements in genome-wide DNA markers and genome sequencing, it has become possible to precisely reveal the mechanisms by which genes regulate phenotypes at the molecular level. In this study, we proposed 447 putative candidate genes associated with candidate SNP markers that were tightly associated with environmental variables. We annotated these genes and found that although these genes do not directly show sensitivity to temperature, precipitation, or ultraviolet radiation, these proteins are involved in maintaining the stability of cell membranes, capturing reactive oxygen species, synthesizing antioxidants, accumulating and regulating osmotic substances, and they have shown good performance in stress resistance potential (Fig. S6B). Fang et al. (2014) identified that the SNP markers associated with temperature adaptation were located in genes such as *HvCbf4B*, *HvPpd-H1*, and *HvVrn-H1* through the *F*st outlier methods. Russell et al. (2016) detected several SNPs near the flowering-related genes such as *HvPRR59*, *HvELF4*, and *HvCO1*. Contreras-Moreira et al. (2019) identified two SNP markers associated with temperature that were located in the well-known cluster of cold acclimation *CBF* genes. In this study, a locus at 67,766,605 bp on chromosome 7H associated with precipitation was found to be located 34 kb downstream of *HvSs1* (Table 1 and Fig. 3A), a known gene induced by cold temperatures. In addition, eight SNPs were located near cold stress-related genes or flowering-regulating genes. For example, the loci at 600,493,261 bp and 600,578,658 bp on chromosome 5H were located not only 1.3 Mb and 1.4 Mb downstream of *HvVrn-H1* but also 0.4 Mb downstream of *IDI7* (Table 1 and Fig. 3C). The locus (7H: 38,883,869) was located 0.8 Mb upstream of *HvVrn-H3* (Table 1 and Fig. 3B). Caproni et al. (2023) believed that loci related to flowering time could also explain environmental adaptation. These results indicated that cold stress-related genes and flowering-regulating genes might play an important role in the naked barley population, and should be given priority consideration in studying the adaptive differentiation of naked barley on QTP. We believe that these data reveal preliminary insights into identification of major putative candidate genes for adaptation to major climate variables on QTP.

## Conclusion

In this study, we explored the climatic factors driving the genetic differentiation of 157 naked barley landraces using 3123 SNPs in a genotyping-by-sequencing assay. Using the pRDA model, we revealed the significant role of natural selection (geographic isolation and climatic factors) in shaping the population structure of the 157 naked barley landraces. By applying outlier methods to 37,636 SNP markers, we identified potential signatures contributing to local adaptation. Within the naked barley landraces, we identified 136 significant loci associated with temperature, precipitation, and ultraviolet radiation, of which 68 (50%) were located in intergenic regions and 13 had pleiotropic effects. Furthermore, we mapped 447 genes, including a known cold stress-responsive gene *HvSs1*. Genes involved in cold stress and flowering time regulation were found in proximity to eight SNP markers. The findings of this study contribute to a better understanding of the genetic mechanisms underlying adaptive traits and provide potential markers for marker-assisted selection of important traits in naked barley breeding. The identification of adaptive loci that respond to environmental factors through landscape genomics demonstrated how landscape genomics could help explain genetic components and adaptive processes. In the near future, whole-genome sequencing of naked barley landraces and the development of the naked barley pangenome will greatly facilitate the discovery of adaptive genes. This could contribute to naked barley breeding and germplasm improvement and lay a foundation for discovering the loci that contribute to climate adaptation in wheat and other cereals on QTP. In addition, in the case of global warming, landscape genomics can be used to assess the adaptability potential of naked barley to the future climate. Further, breeders can assess the agricultural biodiversity potential of local varieties to mitigate the impact of climate change, and put forward suggestions for naked barley breeding for local adaptation.

### Supplementary information

Supplementary Tables

## Data Availability

The entire set of original sequence data has been deposited in the Genome Sequence Archive (https://ngdc.cncb.ac.cn/gsa/) in the BIG Data Center at the Beijing Institute of Genomics (BIG), Chinese Academy of Sciences, under accession number CRA007788.
