# Supplementary material for: Landscape genomics reveals adaptive genetic differentiation driven by multiple environmental variables in naked barley on the Qinghai-Tibetan Plateau
Source: Heredity (Edinb). 2023 Nov 8;131(5-6):316–26. doi: 10.1038/s41437-023-00647-0 (PMC10673939; doi:10.1038/s41437-023-00647-0)
Supplement: Supplementary file 1 — Supplementary Tables [file 41437_2023_647_MOESM1_ESM.pdf]

Supplementary Tables

Table S1 The regions and coordinates of 157 naked barley landraces in this study.

Number	Name	Group	Origin	Longitude	Latitude	Altitude (m)	Row type	Spring/Winter
2	xiningbailiulengqingke	group3	Xining,Qinghai	101.778228	36.617144	2263.3	6	Spring
3	xininghongjiaoni	group3	Xining,Qinghai	101.778228	36.617144	2263.3	6	Spring
4	changmanglanqingke(yushu)	group1	Yushu,Qinghai	97.01	33.01	3648	6	Spring
5	erchangsiduanmangbaiqingke(yushu)	group1	Yushu,Qinghai	97.01	33.01	3648	6	Spring
6	lanqingke(yushu)	admixed	Yushu,Qinghai	97.01	33.01	3648	6	Spring
7	changmangbaiqingke(yushu)	group1	Yushu,Qinghai	97.01	33.01	3648	6	Spring
8	erdaomeibaiqingke(chenduo)	group1	Chenduo,Qinghai	97.05	33.22	3840	6	Spring
9	erdaomeiheiqingke(chenduo)	group1	Chenduo,Qinghai	97.05	33.22	3840	6	Spring
10	changmanghongqingke(chenduo)	group1	Chenduo,Qinghai	97.05	33.22	3840	6	Spring
11	baikangqing(chenduo)	group1	Chenduo,Qinghai	97.05	33.22	3840	6	Spring
12	goumangziqingke(chenduo)	group1	Chenduo,Qinghai	97.05	33.22	3840	6	Spring
13	changmangziqingke(chenduo)	group3	Chenduo,Qinghai	97.05	33.22	3840	6	Spring
14	huangyuanlanqingke	group3	Huangyuan,Qinghai	101.243	36.6922	2663.3	6	Spring
15	huangyuanbailiulengqingke	group3	Huangyuan,Qinghai	101.243	36.6922	2663.3	6	Spring
16	huangyuanhuaqingke	group3	Huangyuan,Qinghai	101.243	36.6922	2663.3	6	Spring
17	huangyuanbailangsan	group3	Huangyuan,Qinghai	101.243	36.6922	2663.3	6	Spring
19	huangzhongzaqingke(bailiuleng)	group3	Huangzhong,Qinghai	101.571667	36.500879	2662.9	6	Spring
21	datongzaqingke(hongsileng)	group3	Datong,Qinghai	101.685643	36.926955	2443.9	6	Spring
23	hongqingke(banma)	group3	Banma,Qinghai	100.44	32.56	3560	6	Spring
24	baiqingke(banma)	group3	Banma,Qinghai	100.44	32.56	3560	6	Spring
25	heiqingke(banma)	group3	Banma,Qinghai	100.44	32.56	3560	6	Spring
27	minhesilenglanqingke	group4	Minhe,Qinghai	102.83097	36.320492	1816.6	6	Spring

28	huzhubaiqingke	group3	Huzhu,Qinghai	101.95838	36.843745	2536.3	6	Spring
30	xunhuabaiqingke(hongmai)	group3	Xunhua,Qinghai	102.485646	35.848586	1872.9	6	Spring
31	changgoumangbaiqingke(nangqian)	group1	Nangqian,Qinghai	96.29	32.12	3644	6	Spring
32	zikangqing(nangqian)	group1	Nangqian,Qinghai	96.29	32.12	3644	6	Spring
33	erdaomeiziqingke(nangqian)	group1	Nangqian,Qinghai	96.29	32.12	3644	6	Spring
34	goumangbaiqingke(nangqian)	group1	Nangqian,Qinghai	96.29	32.12	3644	6	Spring
36	ledusilengbaidamai	group3	Ledu,Qinghai	102.401725	36.482058	1993.8	6	Spring
37	dulanchangmangbaiqingke	group3	Dulan,Qinghai	98.087178	36.297787	3497	6	Spring
38	guidezaqingke	group3	Guide,Qinghai	101.433298	36.04015	2232.9	6	Spring
39	qingke(maqin)	group3	Maqin,Qinghai	100.239015	34.47747	3721.6	6	Null
40	hualongchangmangqingke	group3	Hualong,Qinghai	102.264143	36.094908	2839.9	6	Spring
42	dulihuang	group4	Gannan,Gansu	102.911262	34.983296	2895.2	6	Spring
43	abazhouqingke	admixed	Aba,Sichuan	101.42	32.54	3290	6	Null
44	heilaoya	group3	Menyuan,Qinghai	101.622425	37.376082	3200	6	Null
46	lasashiqingke	group1	Lasa,Tibet	91.140856	29.645554	3656	6	Null
47	jiangzigalalan	group4	Jiangzi,Tibet	89.605574	28.91310649	4030.4	6	Null
102	liulengduanmangqingke(sichuan)	group1	Ganzi,Sichuan	101.962311	30.04952	2517.6	6	Null
104	ganziqingke(sichuan)	group1	Ganzi,Sichuan	101.962311	30.04952	2517.6	6	Spring
109	qianning(sichuan)	group1	Daofu,Sichuan	101.125237	30.979545	3860	6	Spring
111	changduqingke	group1	Changdu,Tibet	97.17202	31.140969	3251.6	6	Spring
112	tewu	group1	Changdu,Tibet	97.17202	31.140969	3251.6	6	Spring
113	mali	group2	Changdu,Tibet	97.17202	31.140969	3251.6	6	Null
116	luolongzong	group1	Luolong,Tibet	95.824601	30.741523	3638	6	Spring
117	shuobanduo	group1	Luolong,Tibet	95.824601	30.741523	3638	6	Spring
118	bianbaqingke	group2	Bianba,Tibet	94.7078	30.933652	3706	6	Spring
119	tuqika	group2	Changdu,Tibet	97.17202	31.140969	3251.6	6	Spring

120	laluqingke	group2	Gongbujiangda,Tibet	93.124088	30.19911192	3980.7	6	Null
121	taizhaoqingke	group1	Linzhi,Tibet	93.246077	29.88528	3426	6	Spring
123	mozhuqingke	group1	Mozhugongka,Tibet	91.730866	29.834132	3840	6	Null
125	lasaqingke	group2	Lasa,Tibet	91.140856	29.645554	3656	6	Spring
127	qushuiqingke	group1	Qushui,Tibet	90.743853	29.353059	3750	6	Spring
128	baidiqingke	group1	Langkazibaidi,Tibet	90.355118	29.112863	4456.4	6	Null
129	nanggazizong	admixed	Linzhi,Tibet	93.074702	29.046337	2995.8	6	Null
133	luobuqingke	group1	Rikaze,Tibet	88.52	29.15	3860	6	Spring
134	bannagangqingke	group2	Rikaze,Tibet	88.52	29.15	3860	6	Spring
135	jiaqingzezaoshuqingke	group1	Rikaze,Tibet	88.52	29.15	3860	6	Null
136	kuxi	group3	Linzhi,Tibet	94.36149	29.649128	2995.8	6	Null
137	baiyu	group2	Bomi,Tibet	95.767913	29.86058765	2744.6	6	Null
139	zhabulai	group1	Yajiang,Sichuan	101.014425	30.031533	2598.6	6	Null
140	handiziqingke	group1	Ganzi,Sichuan	101.962311	30.04952	2517.6	6	Null
141	zumuzuchunqingke	group4	Maerkang,Sichuan	102.206504	31.905813	2640.9	6	Null
142	xiaojinshalonggouwei	admixed	Xiaojin,Sichuan	102.364373	30.999031	2297.9	6	Null
143	danbaheiqingke	admixed	Danba,Sichuan	101.890632	30.878628	2098	6	Spring
144	wumai	group1	Daofu,Sichuan	101.125237	30.979545	3860	6	Null
145	zumuzubaerbali	group4	Daofu,Sichuan	101.125237	30.979545	3860	6	Null
146	ganziheiliuleng	group1	Ganzi,Sichuan	101.962311	30.04952	2517.6	6	Spring
147	daimaoqingke	admixed	Daofu,Sichuan	101.125237	30.979545	3860	6	Spring
148	baiyuqingke	group4	Baiyu,Sichuan	98.824182	31.209913	3983	6	Null
149	ganzibailiuleng	admixed	Ganzi,Sichuan	101.962311	30.04952	2517.6	6	Null
171	luquziqingke	group4	Luqu,Gansu	102.488923	34.590419	3120.7	6	Spring
172	dangchangqingke(lan)	admixed	Dangchang,Gansu	104.393688	34.04699	1857.1	6	Spring
175	daqingke	group3	Linxia,Gansu	103.210539	35.601182	1888.7	6	Null

176	xiaoqingke(jinta)	group3	Jinta,Gansu	98.905591	39.982736	1272	6	Spring
177	wupiqingke	group4	Lixian,Gansu	105.178486	34.189	1415	6	Spring
178	walanqingke	group3	Tianzhu,Gansu	103.14182	36.971812	2397.7	6	Spring
180	changshenziqingke	group4	Lintan,Gansu	103.353133	34.692658	2776.1	6	Spring
182	liulengzichangmangbaiqingke	group3	Yongchang,Gansu	101.973054	38.246914	1958.4	6	Spring
184	liulengziqingke	group3	Lintan,Gansu	103.353133	34.692658	2776.1	6	Spring
185	liulengtoubaiqingke	group2	Gulang,Gansu	102.897544	37.470189	2070.3	6	Spring
186	lijiazhuangliuleng	group3	Lintan,Gansu	103.353133	34.692658	2776.1	6	Spring
199	huangyuanziqingke	group3	Huangyuan,Qinghai	101.243	36.6922	2663.3	6	Spring
200	huzhuheiqingke	group3	Huzhu,Qinghai	101.95838	36.843745	2536.3	6	Spring
201	huzhuheichangmang	group3	Huzhu,Qinghai	101.95838	36.843745	2536.3	6	Spring
203	huazhuziqingke	group3	Huzhu,Qinghai	101.95838	36.843745	2536.3	6	Spring
204	datongzisilengqingke	group3	Datong,Qinghai	101.685643	36.926955	2443.9	6	Spring
205	datongheiqingke	group3	Datong,Qinghai	101.685643	36.926955	2443.9	6	Spring
206	leduheiqingke	group3	Ledu,Qinghai	102.401725	36.482058	1993.8	6	Spring
207	leduhongjiaoni-1	group3	Ledu,Qinghai	102.401725	36.482058	1993.8	6	Spring
208	hualongzisileng	group3	Hualong,Qinghai	102.264143	36.094908	2839.9	6	Spring
209	hualonghongjiaonisileng	group3	Hualong,Qinghai	102.264143	36.094908	2839.9	6	Spring
212	xunhuashandiheiqingke	group3	Xunhua,Qinghai	102.485646	35.848586	1872.9	6	Spring
214	xunhuahongqingke	group3	Xunhua,Qinghai	102.485646	35.848586	1872.9	6	Spring
215	menyuanheilaoya	group3	Menyuan,Qinghai	101.622425	37.376082	3200	6	Spring
217	hainanziqingke	admixed	Gonghe,Qinghai	100.620031	36.284107	2863.8	6	Spring
218	delinghaqingke	group3	Wulan,Qinghai	98.480195	36.929749	2971	6	Spring
220	huangyuanheiliulengqingke	group3	Huangyuan,Qinghai	101.243	36.6922	2663.3	6	Spring
221	datongheiliulengqingke	group3	Datong,Qinghai	101.685643	36.926955	2443.9	6	Spring
224	xunhuahongliulengheiqingke	group3	Xunhua,Qinghai	102.485646	35.848586	1872.9	6	Spring

229	rongqingke	group1	Sangri,Tibet	92.015818	29.26072947	3574.2	2	Null
230	chunqingke	group1	Lasa,Tibet	90.164524	29.431832	3819.8	6	Null
277	xingqingke	group2	Lasa,Tibet	91.140856	29.645554	3656	6	Null
279	lasaqingke(wu)	group2	Lasa,Tibet	91.140856	29.645554	3656	6	Null
280	xueduiqingke	group2	Rikaze,Tibet	89.099243	29.682331	3998.1	6	Null
281	bailangzong	group2	Rikaze,Tibet	88.52	29.15	3860	6	Null
282	dagelazaqingke	group1	Longzi,Tibet	92.461662	28.406809	3873.9	6	Null
362	yuzhongqingke(lan)	group4	Yuzhong,Gansu	104.112527	35.843056	1978.6	6	Spring
366	shihulubaiqingke	group1	Yongchang,Gansu	101.973054	38.246914	1958.4	6	Spring
367	damaqingke	group4	Lintao,Gansu	103.860413	35.374946	1887	6	Spring
370	wuyeerqingke	group4	Gannan,Gansu	102.911262	34.983296	2895.2	6	Spring
371	wuyeerziqingke	group4	Xiahe,Gansu	102.521737	35.202563	2928	6	Spring
372	liulengtou	group3	Yongdeng,Gansu	103.274	36.721	2107	6	Spring
374	liulengtouziqingke	group3	Minle,Gansu	100.812745	38.430635	2305.1	6	Spring
376	liulengqingke	group4	Xihe,Gansu	105.298756	34.014215	1634	6	Spring
379	liulengqingke	group4	Hezheng,Gansu	103.350997	35.424603	2141.4	6	Spring
380	liulengqingke	group4	Zhuoni,Gansu	103.507109	34.589588	2549.5	6	Spring
381	liulenglanqingke	admixed	Minle,Gansu	100.812745	38.430635	2305.1	6	Spring
382	shuangyaoqingke	group4	Minxian,Gansu	104.036832	34.438077	2315.3	6	Spring
383	bendimaqingke	group4	Weiyuan,Gansu	104.215396	35.136794	2088.7	6	Spring
385	silengqingke	group2	Minqin,Gansu	103.093773	38.624406	1376	6	Winter
386	baipiqingke	group3	Zhangye,Gansu	100.449822	38.925646	1482	6	Spring
387	bailansan	group3	Tianzhu,Gansu	103.14182	36.971812	2397.7	6	Spring
389	baiqingke	group3	Jiuquan,Gansu	98.494411	39.732488	1484	6	Spring
390	baiqingke	group3	Yumen,Gansu	97.045535	40.291849	1503.1	6	Spring
391	baiqingke	group3	Minle,Gansu	100.812745	38.430635	2305.1	6	Spring

395	laoqingke	group4	Xihe,Gansu	105.298756	34.014215	1634	6	Spring
396	huimangbaiqingke	group1	Gulang,Gansu	102.897544	37.470189	2070.3	6	Winter
399	qingpiqingke	admixed	Hexi,Gansu	96.850557	40.46474	1597	6	Spring
404	qingke	group4	Tianshui,Gansu	105.724947	34.580862	1173	6	Spring
407	qingke	group4	Yongjing,Gansu	103.315838	35.941685	1661.8	6	Spring
408	qingke	group4	Anxi,Gansu	95.7783167	40.5162837	1178	6	Spring
409	qingke	group3	Wuwei,Gansu	102.638009	37.928336	1532.9	6	Semi-winter
413	ziqingke	group4	Xiahe,Gansu	102.521737	35.202563	2928	6	Spring
414	heiqingke	group3	Yongchang,Gansu	101.973054	38.246914	1958.4	6	Spring
418	lanqingke	group3	Minle,Gansu	100.812745	38.430635	2305.1	6	Spring
420	luodamai	group3	Yumen,Gansu	97.045535	40.291849	1503.1	6	Spring
421	heqingke	group3	Jingtai,Gansu	104.056	37.1944	1637.9	6	Spring
422	manigouliuleng	group3	Linxia,Gansu	103.210539	35.601182	1888.7	6	Spring
426	maqingke	group4	Kangle,Gansu	103.708727	35.369843	2008	6	Spring
428	ziqingke	admixed	Zhuoni,Gansu	103.507109	34.589588	2549.5	6	Spring
438	nuoqingke	group2	Weixi,Yunnan	99.287173	27.177162	2282.1	6	Null
439	songhuamai	group2	Longling,Yunnan	98.689163	24.586695	1556	6	Null
455	xiaozhongdianchangheiqingke	group1	Xiaozhongdian,Yunnan	99.812819	27.553217	3219.6	6	Null
456	nixijiuge	group1	Nixi,Yunan	99.506502	28.04752609	3153.3	6	Null
458	nixibashitianqingke	group1	Nixi,Yunan	99.506502	28.04752609	3153.3	6	Null
460	baiqingke	group2	Deqin,Yunnan	98.911542	28.48611	3278.8	6	Null
703	ziqingke	group1	Yadong,Tibet	88.90703	27.484819	4200	6	Null
704	baizhengliuleng	group1	Jiangzi,Tibet	89.605574	28.91310649	4030.4	6	Null
705	changmangqingke	group1	Milin,Tibet	94.213457	29.215695	2933	6	Null
706	bendi	group1	Renbu,Tibet	89.841993	29.230985	3700	6	Null
707	baijinzi	group1	Naidong,Tibet	91.761539	29.22646087	3559.6	6	Null

708	yangsungabu	group1	Qusong,Tibet	92.203739	29.06438047	3939.4	6	Null
710	xitong	group1	Angren,Tibet	87.236005	29.294809	4349.1	6	Null
712	zhujiuma	group1	Sangri,Tibet	92.015818	29.259189	3567	6	Null
714	qiangbubai	group1	Xiementong,Tibet	88.26162	29.432641	3704.3	6	Null
715	gazangqingke	group1	Dingri,Tibet	87.12612	28.658743	4331	6	Null

Table S2 The full name of thirty climate variables in this study.

	Code	The full name of each climate variable
Precipitation	prec4	Precipitation in April
	prec5	Precipitation in May
	prec6	Precipitation in June
	prec7	Precipitation in July
	prec8	Precipitation in August
	prec9	Precipitation in September
Solar radiation	sra4	Solar radiation in April
	sra5	Solar radiation in May
	sra6	Solar radiation in June
	sra7	Solar radiation in July
	sra8	Solar radiation in August
	sra9	Solar radiation in September
Temperature	tavg4	Average temperature in April
	tavg5	Average temperature in May
	tavg6	Average temperature in June
	tavg7	Average temperature in July
	tavg8	Average temperature in August
	tavg9	Average temperature in September
	tmin4	Minimum temperature in April
	tmin5	Minimum temperature in May
	tmin6	Minimum temperature in June
	tmin7	Minimum temperature in July
	tmin8	Minimum temperature in August
	tmin9	Minimum temperature in September
	tmax4	Maximal temperature in April
	tmax5	Maximal temperature in May
	tmax6	Maximal temperature in June
	tmax7	Maximal temperature in July
	tmax8	Maximal temperature in August
	tmax9	Maximal temperature in September

Table S3 Thirty climate variables were used in this study.

Number	prec4	prec5	prec6	prec7	prec8	prec9	srad4	srad5	srad6	srad7	srad8	srad9	tavg4	tavg5	tavg6	tavg7	tavg8	tavg9	tmin4	tmin5	tmin6	tmin7	tmin8	tmin9	tmax4	tmax5	tmax6	tmax7	tmax8	tmax9
2	20	45	60	88	74	57	18511	20754	21478	20829	19194	15993	8.40	12.60	15.60	17.60	17.10	12.70	1.40	5.90	9.00	11.10	10.80	7.00	15.30	19.40	22.10	24.10	23.40	18.40
3	20	45	60	88	74	57	18511	20754	21478	20829	19194	15993	8.40	12.60	15.60	17.60	17.10	12.70	1.40	5.90	9.00	11.10	10.80	7.00	15.30	19.40	22.10	24.10	23.40	18.40
4	16	54	102	112	87	79	18427	20206	19534	19113	18331	16287	3.20	7.30	10.40	12.20	11.60	8.80	-4.30	0.20	3.90	5.80	4.70	2.30	10.60	14.40	16.90	18.60	18.60	15.30
5	16	54	102	112	87	79	18427	20206	19534	19113	18331	16287	3.20	7.30	10.40	12.20	11.60	8.80	-4.30	0.20	3.90	5.80	4.70	2.30	10.60	14.40	16.90	18.60	18.60	15.30
6	16	54	102	112	87	79	18427	20206	19534	19113	18331	16287	3.20	7.30	10.40	12.20	11.60	8.80	-4.30	0.20	3.90	5.80	4.70	2.30	10.60	14.40	16.90	18.60	18.60	15.30
7	16	54	102	112	87	79	18427	20206	19534	19113	18331	16287	3.20	7.30	10.40	12.20	11.60	8.80	-4.30	0.20	3.90	5.80	4.70	2.30	10.60	14.40	16.90	18.60	18.60	15.30
8	19	54	103	120	94	82	18429	20234	19615	19038	18161	16234	-0.20	4.20	7.40	9.30	8.70	5.80	-7.20	-2.40	1.30	3.40	2.30	-0.10	6.80	10.70	13.40	15.10	15.00	11.80
9	19	54	103	120	94	82	18429	20234	19615	19038	18161	16234	-0.20	4.20	7.40	9.30	8.70	5.80	-7.20	-2.40	1.30	3.40	2.30	-0.10	6.80	10.70	13.40	15.10	15.00	11.80
10	19	54	103	120	94	82	18429	20234	19615	19038	18161	16234	-0.20	4.20	7.40	9.30	8.70	5.80	-7.20	-2.40	1.30	3.40	2.30	-0.10	6.80	10.70	13.40	15.10	15.00	11.80
11	19	54	103	120	94	82	18429	20234	19615	19038	18161	16234	-0.20	4.20	7.40	9.30	8.70	5.80	-7.20	-2.40	1.30	3.40	2.30	-0.10	6.80	10.70	13.40	15.10	15.00	11.80
12	19	54	103	120	94	82	18429	20234	19615	19038	18161	16234	-0.20	4.20	7.40	9.30	8.70	5.80	-7.20	-2.40	1.30	3.40	2.30	-0.10	6.80	10.70	13.40	15.10	15.00	11.80
13	19	54	103	120	94	82	18429	20234	19615	19038	18161	16234	-0.20	4.20	7.40	9.30	8.70	5.80	-7.20	-2.40	1.30	3.40	2.30	-0.10	6.80	10.70	13.40	15.10	15.00	11.80
14	20	51	71	97	85	65	18817	20907	21713	21036	19459	16264	4.90	9.10	11.90	13.90	13.50	9.30	-2.20	2.40	5.70	7.60	6.90	3.20	12.00	15.90	18.20	20.30	20.10	15.40
15	20	51	71	97	85	65	18817	20907	21713	21036	19459	16264	4.90	9.10	11.90	13.90	13.50	9.30	-2.20	2.40	5.70	7.60	6.90	3.20	12.00	15.90	18.20	20.30	20.10	15.40
16	20	51	71	97	85	65	18817	20907	21713	21036	19459	16264	4.90	9.10	11.90	13.90	13.50	9.30	-2.20	2.40	5.70	7.60	6.90	3.20	12.00	15.90	18.20	20.30	20.10	15.40
17	20	51	71	97	85	65	18817	20907	21713	21036	19459	16264	4.90	9.10	11.90	13.90	13.50	9.30	-2.20	2.40	5.70	7.60	6.90	3.20	12.00	15.90	18.20	20.30	20.10	15.40
19	22	52	71	99	86	67	18505	20668	21380	20699	19015	15836	5.80	10.00	12.60	14.30	14.00	9.90	-1.40	3.30	6.50	8.20	7.80	4.20	13.10	16.80	18.70	20.40	20.20	15.60
21	21	50	63	92	85	63	18802	20978	21981	21129	19434	16312	5.70	10.00	12.80	14.70	14.30	10.10	-1.20	3.40	6.40	8.30	8.00	4.20	12.70	16.60	19.20	21.20	20.70	16.00
23	37	80	138	133	109	119	16637	18259	17902	17342	16520	14261	-1.40	2.80	6.10	7.70	7.20	4.90	-7.50	-3.30	0.40	2.10	1.20	-1.00	4.80	8.90	11.90	13.40	13.20	10.70
24	37	80	138	133	109	119	16637	18259	17902	17342	16520	14261	-1.40	2.80	6.10	7.70	7.20	4.90	-7.50	-3.30	0.40	2.10	1.20	-1.00	4.80	8.90	11.90	13.40	13.20	10.70
25	37	80	138	133	109	119	16637	18259	17902	17342	16520	14261	-1.40	2.80	6.10	7.70	7.20	4.90	-7.50	-3.30	0.40	2.10	1.20	-1.00	4.80	8.90	11.90	13.40	13.20	10.70
27	19	41	47	71	74	52	17941	20325	21241	20685	19042	15444	10.40	15.40	18.90	21.10	20.20	15.00	3.80	8.70	12.70	14.80	14.70	9.80	17.00	22.00	25.10	27.30	25.60	20.20
28	21	48	63	91	85	63	18606	20831	21766	21003	19279	16068	5.70	10.00	12.70	14.50	14.10	10.10	-1.40	3.30	6.50	8.40	8.10	4.40	12.70	16.70	18.90	20.60	20.20	15.80
30	24	51	59	85	85	65	17729	20027	20753	20461	18826	15227	10.30	14.90	18.10	20.20	19.50	14.70	3.70	8.20	11.70	13.80	13.40	9.20	16.90	21.60	24.60	26.60	25.50	20.10

31	21	56	115	133	108	92	18104	19986	18931	18032	17413	16181	-1.10	3.30	6.90	8.50	8.20	5.80	-7.30	-2.70	1.00	2.90	2.20	0.00	5.20	9.30	12.90	14.10	14.10	11.70
32	21	56	115	133	108	92	18104	19986	18931	18032	17413	16181	-1.10	3.30	6.90	8.50	8.20	5.80	-7.30	-2.70	1.00	2.90	2.20	0.00	5.20	9.30	12.90	14.10	14.10	11.70
33	21	56	115	133	108	92	18104	19986	18931	18032	17413	16181	-1.10	3.30	6.90	8.50	8.20	5.80	-7.30	-2.70	1.00	2.90	2.20	0.00	5.20	9.30	12.90	14.10	14.10	11.70
34	21	56	115	133	108	92	18104	19986	18931	18032	17413	16181	-1.10	3.30	6.90	8.50	8.20	5.80	-7.30	-2.70	1.00	2.90	2.20	0.00	5.20	9.30	12.90	14.10	14.10	11.70
36	18	41	49	74	72	53	18264	20549	21555	20991	19320	15784	9.50	14.10	17.30	19.40	18.90	13.90	2.70	7.40	10.90	13.00	13.10	8.40	16.20	20.70	23.70	25.70	24.60	19.30
37	9	24	42	46	28	16	20475	22085	22645	22271	20540	17601	4.10	9.10	12.40	14.80	14.30	9.60	-2.90	2.40	6.20	8.60	7.80	3.00	11.00	15.80	18.70	21.10	20.90	16.10
38	21	51	71	93	80	64	18295	20440	21041	20726	19202	15951	8.70	12.70	15.60	17.60	17.60	13.00	1.80	6.10	9.10	11.10	10.90	6.90	15.60	19.30	22.00	24.10	24.20	19.10
39	25	60	99	111	90	80	17804	19614	19673	19292	18046	15366	1.00	4.90	7.90	9.80	9.50	6.10	-6.50	-1.60	1.90	3.80	2.80	-0.10	8.50	11.50	14.00	15.90	16.10	12.40
40	25	56	73	101	93	72	17947	20106	20770	20237	18734	15368	4.90	9.10	12.10	14.00	13.50	9.40	-2.20	2.50	6.10	8.20	7.60	4.00	11.90	15.70	18.20	19.80	19.40	14.80
42	34	71	80	111	108	92	16955	19047	19756	19333	17974	14369	4.30	8.30	11.20	13.30	12.90	9.10	-2.70	1.50	5.00	7.20	6.40	3.30	11.40	15.00	17.40	19.50	19.40	14.90
43	43	90	145	128	105	126	16014	17737	17452	17255	16537	13748	3.10	6.80	9.50	11.10	10.80	8.20	-3.70	0.40	3.60	5.10	4.40	2.40	9.90	13.30	15.50	17.10	17.30	14.00
44	27	58	78	102	104	75	18937	21197	22110	21247	19511	16540	2.10	6.70	9.80	11.60	11.00	7.20	-5.00	-0.30	3.20	5.10	4.30	0.80	9.30	13.70	16.40	18.20	17.70	13.50
46	6	26	71	117	118	66	20543	22196	20688	18567	18402	17616	7.80	11.80	15.30	15.20	14.30	12.40	0.40	4.70	8.50	9.10	8.60	6.50	15.10	18.90	22.00	21.20	20.00	18.30
47	3	16	47	87	91	39	20557	21749	20734	18777	18656	17405	4.80	8.80	12.30	12.40	11.70	10.30	-3.20	1.30	5.80	6.90	6.40	4.30	12.70	16.40	18.70	18.00	17.10	16.30
102	39	83	158	143	115	128	15231	16345	15871	15368	15412	12695	10.40	13.70	15.90	17.80	17.60	14.30	4.50	8.10	11.20	13.20	12.70	10.00	16.40	19.20	20.50	22.50	22.50	18.60
104	39	83	158	143	115	128	15231	16345	15871	15368	15412	12695	10.40	13.70	15.90	17.80	17.60	14.30	4.50	8.10	11.20	13.20	12.70	10.00	16.40	19.20	20.50	22.50	22.50	18.60
109	26	63	130	114	101	108	15712	17095	16726	16201	15971	13678	9.40	12.90	15.20	16.40	16.20	13.90	1.10	5.30	8.60	9.90	9.20	7.20	17.60	20.60	21.90	22.90	23.30	20.60
111	22	43	98	110	96	81	17299	19346	18161	17838	17448	16019	7.60	11.80	15.00	15.90	15.20	12.80	0.20	4.40	8.00	9.30	8.30	5.90	14.90	19.30	22.10	22.50	22.00	19.60
112	22	43	98	110	96	81	17299	19346	18161	17838	17448	16019	7.60	11.80	15.00	15.90	15.20	12.80	0.20	4.40	8.00	9.30	8.30	5.90	14.90	19.30	22.10	22.50	22.00	19.60
113	22	43	98	110	96	81	17299	19346	18161	17838	17448	16019	7.60	11.80	15.00	15.90	15.20	12.80	0.20	4.40	8.00	9.30	8.30	5.90	14.90	19.30	22.10	22.50	22.00	19.60
116	31	57	117	134	108	97	17973	19651	18309	17341	17087	16047	4.80	9.10	12.60	13.40	13.00	10.90	-1.50	2.50	6.30	7.50	6.90	5.10	11.20	15.60	18.80	19.30	19.10	16.70
117	31	57	117	134	108	97	17973	19651	18309	17341	17087	16047	4.80	9.10	12.60	13.40	13.00	10.90	-1.50	2.50	6.30	7.50	6.90	5.10	11.20	15.60	18.80	19.30	19.10	16.70
118	29	62	125	133	103	100	18670	20291	19094	18145	17750	16777	4.50	8.40	11.90	13.00	12.70	10.40	-1.90	2.10	6.00	7.30	6.60	4.60	10.80	14.80	17.80	18.70	18.70	16.20
119	22	43	98	110	96	81	17299	19346	18161	17838	17448	16019	7.60	11.80	15.00	15.90	15.20	12.80	0.20	4.40	8.00	9.30	8.30	5.90	14.90	19.30	22.10	22.50	22.00	19.60
120	22	52	103	121	102	85	19410	20864	19407	18061	17875	16833	3.00	7.00	10.50	11.40	11.10	9.00	-3.00	1.20	4.90	6.20	6.00	4.10	8.90	12.80	16.10	16.60	16.30	14.00
121	20	46	94	116	97	82	19358	20644	19244	18100	17956	16905	6.40	9.80	13.00	13.90	13.40	11.70	0.00	3.60	7.10	8.40	7.80	6.50	12.80	16.00	18.90	19.40	19.00	17.00

123	6	25	68	108	101	62	20349	21878	20852	19035	18873	17840	5.50	9.60	13.20	13.30	12.60	10.70	-2.00	2.50	6.70	7.50	6.90	5.00	13.00	16.70	19.70	19.10	18.30	16.40
125	6	26	71	117	118	66	20543	22196	20688	18567	18402	17616	7.80	11.80	15.30	15.20	14.30	12.40	0.40	4.70	8.50	9.10	8.60	6.50	15.10	18.90	22.00	21.20	20.00	18.30
127	7	26	65	110	118	64	20571	21985	21009	19117	18841	17665	8.30	12.30	15.70	15.50	14.70	12.90	0.90	5.20	9.10	9.70	9.20	7.20	15.70	19.40	22.20	21.30	20.20	18.60
128	6	18	51	88	84	40	20435	21776	20450	18451	18279	17136	1.70	5.50	9.30	9.50	8.70	7.00	-6.10	-1.60	2.80	4.10	3.70	1.50	9.50	12.60	15.70	14.90	13.80	12.50
129	19	37	77	114	105	74	19520	20363	18976	17922	17782	16975	8.50	12.00	15.00	15.80	15.20	13.60	2.00	6.00	9.40	10.50	10.00	8.30	15.00	18.10	20.70	21.00	20.40	18.80
133	4	18	52	111	128	54	20629	21820	20527	18753	18238	17074	5.10	9.10	13.10	12.90	12.00	10.60	-3.00	1.20	5.90	6.90	6.30	4.20	13.10	17.00	20.20	18.90	17.70	16.90
134	4	18	52	111	128	54	20629	21820	20527	18753	18238	17074	5.10	9.10	13.10	12.90	12.00	10.60	-3.00	1.20	5.90	6.90	6.30	4.20	13.10	17.00	20.20	18.90	17.70	16.90
135	4	18	52	111	128	54	20629	21820	20527	18753	18238	17074	5.10	9.10	13.10	12.90	12.00	10.60	-3.00	1.20	5.90	6.90	6.30	4.20	13.10	17.00	20.20	18.90	17.70	16.90
136	45	70	123	133	110	112	18657	19769	18203	17360	17244	16201	8.90	12.10	15.10	16.10	15.70	13.90	2.70	6.00	9.70	10.90	10.40	8.80	15.00	18.20	20.50	21.20	21.00	19.00
137	59	78	162	174	150	134	17815	19159	17390	16596	16449	15114	8.00	11.80	14.70	15.90	15.70	13.80	2.40	6.00	9.20	10.90	10.40	8.90	13.70	17.60	20.20	20.80	20.90	18.80
139	29	62	141	146	117	118	15821	16889	16277	15528	15600	13480	10.60	14.50	16.70	17.90	17.50	15.20	4.30	8.60	11.50	12.90	12.10	10.40	16.80	20.30	21.90	22.90	22.90	19.90
140	39	83	158	143	115	128	15231	16345	15871	15368	15412	12695	10.40	13.70	15.90	17.80	17.60	14.30	4.50	8.10	11.20	13.20	12.70	10.00	16.40	19.20	20.50	22.50	22.50	18.60
141	42	86	127	107	84	110	15298	16970	17001	16710	16234	13255	9.70	12.90	15.10	16.70	16.50	13.50	2.20	5.80	8.50	10.30	9.50	7.30	17.20	19.90	21.60	23.10	23.50	19.80
142	42	88	131	96	71	101	14925	16378	16328	16030	15663	12924	12.20	15.30	16.90	19.00	18.90	15.70	5.20	8.70	11.30	13.50	12.80	10.30	19.20	21.80	22.50	24.50	25.00	21.10
143	38	78	134	127	106	113	15106	16592	16339	16012	15718	13079	14.70	18.00	19.70	21.60	21.40	18.30	7.80	11.90	14.30	16.30	15.60	13.30	21.50	24.10	25.10	26.90	27.10	23.30
144	26	63	130	114	101	108	15712	17095	16726	16201	15971	13678	9.40	12.90	15.20	16.40	16.20	13.90	1.10	5.30	8.60	9.90	9.20	7.20	17.60	20.60	21.90	22.90	23.30	20.60
145	26	63	130	114	101	108	15712	17095	16726	16201	15971	13678	9.40	12.90	15.20	16.40	16.20	13.90	1.10	5.30	8.60	9.90	9.20	7.20	17.60	20.60	21.90	22.90	23.30	20.60
146	39	83	158	143	115	128	15231	16345	15871	15368	15412	12695	10.40	13.70	15.90	17.80	17.60	14.30	4.50	8.10	11.20	13.20	12.70	10.00	16.40	19.20	20.50	22.50	22.50	18.60
147	26	63	130	114	101	108	15712	17095	16726	16201	15971	13678	9.40	12.90	15.20	16.40	16.20	13.90	1.10	5.30	8.60	9.90	9.20	7.20	17.60	20.60	21.90	22.90	23.30	20.60
148	25	52	112	121	101	97	16903	18520	17791	17328	16841	15052	7.60	11.60	14.40	15.40	14.80	12.70	0.90	4.90	8.00	9.30	8.60	6.60	14.20	18.20	20.70	21.50	21.10	18.80
149	39	83	158	143	115	128	15231	16345	15871	15368	15412	12695	10.40	13.70	15.90	17.80	17.60	14.30	4.50	8.10	11.20	13.20	12.70	10.00	16.40	19.20	20.50	22.50	22.50	18.60
171	35	75	92	119	110	99	16826	18817	19364	18968	17791	14340	3.80	7.40	10.10	12.10	11.80	8.20	-3.20	0.80	4.20	6.10	5.50	2.50	10.70	14.00	16.10	18.10	18.10	14.00
172	46	80	79	108	100	93	15863	18092	19156	18881	17596	13135	9.50	13.50	16.60	18.60	17.90	13.60	3.70	7.50	11.10	13.20	12.60	9.00	15.30	19.40	22.00	24.00	23.20	18.20
175	34	61	58	90	100	75	17234	19599	20469	20018	18453	14578	10.10	14.60	17.70	19.70	18.90	14.20	3.50	8.10	11.60	13.70	13.20	9.00	16.70	21.10	23.70	25.60	24.50	19.30
176	4	9	16	23	21	11	20490	22918	24144	23455	21740	18501	9.30	15.50	19.90	22.00	20.60	15.00	1.70	7.90	12.60	14.80	13.30	7.50	17.00	23.10	27.30	29.30	28.00	22.60
177	47	77	83	116	105	97	16067	18333	19366	18871	17498	13118	11.00	15.20	18.80	20.80	19.80	15.20	5.30	9.30	13.10	15.40	14.60	10.70	16.70	21.00	24.50	26.10	25.00	19.70

178	16	34	49	69	72	48	18290	20703	21543	20666	19103	15780	5.60	10.80	14.30	16.50	15.80	10.80	-0.80	4.30	8.20	10.60	10.60	5.60	12.10	17.20	20.50	22.50	21.00	15.90
180	39	74	81	110	108	93	16601	18735	19466	19118	17806	14128	5.30	9.20	12.00	14.00	13.60	9.70	-1.60	2.60	6.00	8.20	7.70	4.30	12.20	15.90	18.00	19.80	19.50	15.00
182	11	25	38	48	55	37	19464	21902	22862	22087	20363	17283	6.00	10.90	13.90	16.10	16.00	11.40	-1.30	3.90	7.30	9.40	9.50	4.70	13.30	17.80	20.60	22.80	22.60	18.20
184	39	74	81	110	108	93	16601	18735	19466	19118	17806	14128	5.30	9.20	12.00	14.00	13.60	9.70	-1.60	2.60	6.00	8.20	7.70	4.30	12.20	15.90	18.00	19.80	19.50	15.00
185	12	26	36	50	58	38	18782	21240	22391	21465	19775	16496	6.70	12.00	15.80	18.10	17.80	12.40	-0.10	5.40	9.10	11.50	12.40	6.50	13.50	18.60	22.50	24.60	23.20	18.20
186	39	74	81	110	108	93	16601	18735	19466	19118	17806	14128	5.30	9.20	12.00	14.00	13.60	9.70	-1.60	2.60	6.00	8.20	7.70	4.30	12.20	15.90	18.00	19.80	19.50	15.00
199	20	51	71	97	85	65	18817	20907	21713	21036	19459	16264	4.90	9.10	11.90	13.90	13.50	9.30	-2.20	2.40	5.70	7.60	6.90	3.20	12.00	15.90	18.20	20.30	20.10	15.40
200	21	48	63	91	85	63	18606	20831	21766	21003	19279	16068	5.70	10.00	12.70	14.50	14.10	10.10	-1.40	3.30	6.50	8.40	8.10	4.40	12.70	16.70	18.90	20.60	20.20	15.80
201	21	48	63	91	85	63	18606	20831	21766	21003	19279	16068	5.70	10.00	12.70	14.50	14.10	10.10	-1.40	3.30	6.50	8.40	8.10	4.40	12.70	16.70	18.90	20.60	20.20	15.80
203	21	48	63	91	85	63	18606	20831	21766	21003	19279	16068	5.70	10.00	12.70	14.50	14.10	10.10	-1.40	3.30	6.50	8.40	8.10	4.40	12.70	16.70	18.90	20.60	20.20	15.80
204	21	50	63	92	85	63	18802	20978	21981	21129	19434	16312	5.70	10.00	12.80	14.70	14.30	10.10	-1.20	3.40	6.40	8.30	8.00	4.20	12.70	16.60	19.20	21.20	20.70	16.00
205	21	50	63	92	85	63	18802	20978	21981	21129	19434	16312	5.70	10.00	12.80	14.70	14.30	10.10	-1.20	3.40	6.40	8.30	8.00	4.20	12.70	16.60	19.20	21.20	20.70	16.00
206	18	41	49	74	72	53	18264	20549	21555	20991	19320	15784	9.50	14.10	17.30	19.40	18.90	13.90	2.70	7.40	10.90	13.00	13.10	8.40	16.20	20.70	23.70	25.70	24.60	19.30
207	18	41	49	74	72	53	18264	20549	21555	20991	19320	15784	9.50	14.10	17.30	19.40	18.90	13.90	2.70	7.40	10.90	13.00	13.10	8.40	16.20	20.70	23.70	25.70	24.60	19.30
208	25	56	73	101	93	72	17947	20106	20770	20237	18734	15368	4.90	9.10	12.10	14.00	13.50	9.40	-2.20	2.50	6.10	8.20	7.60	4.00	11.90	15.70	18.20	19.80	19.40	14.80
209	25	56	73	101	93	72	17947	20106	20770	20237	18734	15368	4.90	9.10	12.10	14.00	13.50	9.40	-2.20	2.50	6.10	8.20	7.60	4.00	11.90	15.70	18.20	19.80	19.40	14.80
212	24	51	59	85	85	65	17729	20027	20753	20461	18826	15227	10.30	14.90	18.10	20.20	19.50	14.70	3.70	8.20	11.70	13.80	13.40	9.20	16.90	21.60	24.60	26.60	25.50	20.10
214	24	51	59	85	85	65	17729	20027	20753	20461	18826	15227	10.30	14.90	18.10	20.20	19.50	14.70	3.70	8.20	11.70	13.80	13.40	9.20	16.90	21.60	24.60	26.60	25.50	20.10
215	27	58	78	102	104	75	18937	21197	22110	21247	19511	16540	2.10	6.70	9.80	11.60	11.00	7.20	-5.00	-0.30	3.20	5.10	4.30	0.80	9.30	13.70	16.40	18.20	17.70	13.50
217	15	46	70	89	72	51	18882	20852	21440	20997	19363	16432	5.30	9.80	12.90	15.00	14.40	10.00	-2.30	2.70	6.30	8.40	7.50	3.40	13.00	16.90	19.40	21.60	21.40	16.50
218	9	25	47	47	36	21	20354	22077	22734	22555	20570	17674	3.40	8.80	12.10	14.40	13.90	9.20	-3.80	2.00	5.80	8.10	7.30	2.40	10.70	15.60	18.50	20.60	20.50	15.90
220	20	51	71	97	85	65	18817	20907	21713	21036	19459	16264	4.90	9.10	11.90	13.90	13.50	9.30	-2.20	2.40	5.70	7.60	6.90	3.20	12.00	15.90	18.20	20.30	20.10	15.40
221	21	50	63	92	85	63	18802	20978	21981	21129	19434	16312	5.70	10.00	12.80	14.70	14.30	10.10	-1.20	3.40	6.40	8.30	8.00	4.20	12.70	16.60	19.20	21.20	20.70	16.00
224	24	51	59	85	85	65	17729	20027	20753	20461	18826	15227	10.30	14.90	18.10	20.20	19.50	14.70	3.70	8.20	11.70	13.80	13.40	9.20	16.90	21.60	24.60	26.60	25.50	20.10
229	7	20	55	98	89	52	20188	21367	20183	18554	18406	17429	6.90	10.80	14.40	14.50	13.80	12.20	-0.70	3.60	7.80	8.60	7.90	6.00	14.40	18.00	21.10	20.50	19.70	18.40
230	4	22	62	107	114	57	20635	21994	21040	19269	18891	17630	6.60	10.70	14.30	14.30	13.50	11.90	-1.00	3.30	7.50	8.10	7.70	5.60	14.20	18.00	21.10	20.50	19.30	18.30

277	6	26	71	117	118	66	20543	22196	20688	18567	18402	17616	7.80	11.80	15.30	15.20	14.30	12.40	0.40	4.70	8.50	9.10	8.60	6.50	15.10	18.90	22.00	21.20	20.00	18.30
279	6	26	71	117	118	66	20543	22196	20688	18567	18402	17616	7.80	11.80	15.30	15.20	14.30	12.40	0.40	4.70	8.50	9.10	8.60	6.50	15.10	18.90	22.00	21.20	20.00	18.30
280	3	19	60	124	135	56	20542	21953	21012	18979	18507	17392	4.90	8.90	12.70	12.70	11.90	10.10	-2.80	1.50	5.90	7.00	6.60	4.30	12.60	16.40	19.40	18.50	17.20	15.90
281	4	18	52	111	128	54	20629	21820	20527	18753	18238	17074	5.10	9.10	13.10	12.90	12.00	10.60	-3.00	1.20	5.90	6.90	6.30	4.20	13.10	17.00	20.20	18.90	17.70	16.90
282	10	17	43	83	77	39	19887	20592	19255	17778	17884	17023	5.50	9.60	13.50	13.80	12.90	11.40	-2.50	2.40	7.00	8.00	7.30	5.20	13.40	16.80	20.00	19.50	18.60	17.60
362	26	45	49	75	85	58	17228	19684	20626	20013	18309	14489	8.60	13.30	16.60	18.90	18.00	13.10	2.20	7.00	10.70	13.00	12.20	7.80	15.10	19.60	22.40	24.70	23.70	18.30
366	11	25	38	48	55	37	19464	21902	22862	22087	20363	17283	6.00	10.90	13.90	16.10	16.00	11.40	-1.30	3.90	7.30	9.40	9.50	4.70	13.30	17.80	20.60	22.80	22.60	18.20
367	33	59	58	88	95	71	16938	19315	20276	19881	18280	14261	9.20	13.70	16.70	18.90	18.00	13.40	2.90	7.40	11.00	13.20	12.50	8.50	15.50	19.90	22.50	24.60	23.60	18.40
370	34	71	80	111	108	92	16955	19047	19756	19333	17974	14369	4.30	8.30	11.20	13.30	12.90	9.10	-2.70	1.50	5.00	7.20	6.40	3.30	11.40	15.00	17.40	19.50	19.40	14.90
371	32	67	81	112	104	88	17209	19337	19972	19475	18255	14667	4.20	8.20	11.20	13.40	12.90	9.00	-2.80	1.50	5.10	7.20	6.50	3.20	11.20	14.80	17.40	19.50	19.30	14.70
372	16	34	43	63	70	47	18098	20534	21480	20709	19019	15493	7.90	13.00	16.60	18.80	18.00	12.90	1.10	6.30	10.20	12.60	12.50	7.40	14.70	19.70	23.00	25.00	23.50	18.30
374	13	33	56	77	77	51	19736	22058	23073	22153	20344	17458	4.50	9.90	13.30	15.40	14.70	10.20	-3.00	2.60	6.70	8.60	8.00	3.40	12.00	17.20	19.90	22.10	21.40	17.00
376	48	78	84	117	105	100	15897	18127	19166	18759	17491	13008	10.70	14.80	18.10	20.30	19.40	14.90	5.00	9.00	12.60	15.10	14.30	10.40	16.30	20.60	23.60	25.50	24.60	19.30
379	34	63	61	93	100	78	17122	19403	20361	19890	18425	14403	8.20	12.30	15.10	17.40	16.90	12.40	1.50	5.90	9.30	11.60	11.20	7.30	14.80	18.60	20.90	23.20	22.60	17.40
380	40	76	79	109	106	93	16492	18633	19425	19042	17839	13902	6.20	10.30	12.80	15.00	14.70	10.60	-0.20	3.90	7.10	9.30	8.90	5.60	12.70	16.60	18.60	20.80	20.50	15.60
381	13	33	56	77	77	51	19736	22058	23073	22153	20344	17458	4.50	9.90	13.30	15.40	14.70	10.20	-3.00	2.60	6.70	8.60	8.00	3.40	12.00	17.20	19.90	22.10	21.40	17.00
382	45	80	80	108	106	92	16240	18451	19328	19039	17684	13629	7.40	11.60	14.30	16.50	16.00	11.60	1.20	5.30	8.50	10.90	10.50	7.00	13.60	17.90	20.10	22.10	21.50	16.30
383	36	63	64	93	97	77	16795	19110	20112	19821	18230	14015	7.50	12.00	15.20	17.30	16.40	11.90	1.20	5.70	9.60	11.90	11.10	7.20	13.80	18.30	20.90	22.70	21.70	16.70
385	5	11	15	22	31	17	19667	22282	23559	22705	20899	17827	9.90	15.60	19.30	21.20	21.20	15.60	2.30	8.30	12.00	14.10	14.60	8.70	17.40	22.80	26.60	28.30	27.90	22.60
386	7	19	32	49	48	29	20163	22530	23647	22793	21149	18085	9.40	14.90	18.80	21.10	19.90	14.50	1.70	7.30	11.30	13.70	12.60	6.90	17.10	22.50	26.20	28.50	27.20	22.10
387	16	34	49	69	72	48	18290	20703	21543	20666	19103	15780	5.60	10.80	14.30	16.50	15.80	10.80	-0.80	4.30	8.20	10.60	10.60	5.60	12.10	17.20	20.50	22.50	21.00	15.90
389	4	9	17	22	21	10	20478	22802	24019	23362	21650	18496	9.10	15.10	19.30	21.20	19.90	14.30	1.90	7.90	12.30	14.30	12.70	7.20	16.30	22.30	26.30	28.20	27.00	21.40
390	5	9	11	16	12	6	20552	23122	24273	23515	21753	18629	9.60	15.60	19.70	21.60	20.60	15.30	1.70	7.90	12.30	14.40	13.10	7.50	17.50	23.40	27.10	28.90	28.10	23.00
391	13	33	56	77	77	51	19736	22058	23073	22153	20344	17458	4.50	9.90	13.30	15.40	14.70	10.20	-3.00	2.60	6.70	8.60	8.00	3.40	12.00	17.20	19.90	22.10	21.40	17.00
395	48	78	84	117	105	100	15897	18127	19166	18759	17491	13008	10.70	14.80	18.10	20.30	19.40	14.90	5.00	9.00	12.60	15.10	14.30	10.40	16.30	20.60	23.60	25.50	24.60	19.30
396	12	26	36	50	58	38	18782	21240	22391	21465	19775	16496	6.70	12.00	15.80	18.10	17.80	12.40	-0.10	5.40	9.10	11.50	12.40	6.50	13.50	18.60	22.50	24.60	23.20	18.20

399	5	8	10	15	11	6	20563	23178	24304	23564	21826	18654	9.80	15.90	19.80	21.60	20.60	15.40	1.80	8.10	12.50	14.50	13.20	7.60	17.80	23.80	27.10	28.70	28.00	23.20
404	45	69	74	116	106	100	16640	18966	20116	19444	18065	13524	12.80	17.10	20.70	22.90	22.00	17.00	6.90	11.10	14.80	17.50	16.70	12.40	18.80	23.10	26.60	28.30	27.30	21.60
407	24	46	48	73	82	57	17533	19935	20869	20529	18905	14985	11.10	15.90	19.30	21.20	20.20	15.30	4.60	9.30	13.00	15.10	14.60	10.10	17.60	22.50	25.60	27.30	25.80	20.60
408	4	6	8	13	8	3	20559	23385	24520	23772	22025	18988	11.70	18.00	21.60	23.50	22.40	16.90	3.20	9.70	14.00	16.00	14.80	8.60	20.20	26.40	29.20	31.00	30.00	25.10
409	8	17	26	31	40	26	19213	21728	22820	21802	20217	17075	9.40	14.50	18.20	20.20	20.60	14.60	2.30	7.90	11.20	13.40	15.70	8.50	16.40	21.20	25.20	27.00	25.40	20.80
413	32	67	81	112	104	88	17209	19337	19972	19475	18255	14667	4.20	8.20	11.20	13.40	12.90	9.00	-2.80	1.50	5.10	7.20	6.50	3.20	11.20	14.80	17.40	19.50	19.30	14.70
414	11	25	38	48	55	37	19464	21902	22862	22087	20363	17283	6.00	10.90	13.90	16.10	16.00	11.40	-1.30	3.90	7.30	9.40	9.50	4.70	13.30	17.80	20.60	22.80	22.60	18.20
418	13	33	56	77	77	51	19736	22058	23073	22153	20344	17458	4.50	9.90	13.30	15.40	14.70	10.20	-3.00	2.60	6.70	8.60	8.00	3.40	12.00	17.20	19.90	22.10	21.40	17.00
420	5	9	11	16	12	6	20552	23122	24273	23515	21753	18629	9.60	15.60	19.70	21.60	20.60	15.30	1.70	7.90	12.30	14.40	13.10	7.50	17.50	23.40	27.10	28.90	28.10	23.00
421	10	19	26	40	52	28	18421	21049	22256	21480	19687	16298	9.10	14.50	18.10	19.90	19.40	14.80	2.00	7.60	11.40	13.50	13.70	8.50	16.30	21.40	24.80	26.30	25.10	21.00
422	34	61	58	90	100	75	17234	19599	20469	20018	18453	14578	10.10	14.60	17.70	19.70	18.90	14.20	3.50	8.10	11.60	13.70	13.20	9.00	16.70	21.10	23.70	25.60	24.50	19.30
426	33	60	58	88	95	73	17018	19341	20342	20045	18514	14314	8.60	13.00	16.10	18.30	17.40	12.80	2.10	6.60	10.10	12.40	11.80	7.80	15.20	19.40	22.00	24.10	23.00	17.80
428	40	76	79	109	106	93	16492	18633	19425	19042	17839	13902	6.20	10.30	12.80	15.00	14.70	10.60	-0.20	3.90	7.10	9.30	8.90	5.60	12.70	16.60	18.60	20.80	20.50	15.60
438	107	42	98	157	154	99	18211	17825	15518	14072	14788	14172	12.20	16.20	18.70	19.20	18.70	17.20	6.50	10.70	14.50	15.20	14.40	13.10	18.00	21.80	23.00	23.20	23.00	21.30
439	58	120	238	293	258	161	20502	19179	15950	13842	14946	15799	17.90	20.30	21.60	21.50	21.70	20.90	11.10	15.00	18.10	18.40	18.00	16.70	24.80	25.70	25.10	24.50	25.30	25.00
455	72	47	91	138	123	88	17453	17310	15629	14128	14689	13824	6.20	10.70	13.60	14.20	13.70	12.20	-0.40	4.30	8.60	9.70	9.10	7.60	12.80	17.20	18.60	18.70	18.40	16.90
456	71	43	86	137	119	83	17300	17417	15877	14408	14848	13933	5.90	10.70	13.70	14.20	13.60	12.30	0.20	4.80	8.90	9.80	9.10	7.80	11.60	16.50	18.50	18.50	18.10	16.80
458	71	43	86	137	119	83	17300	17417	15877	14408	14848	13933	5.90	10.70	13.70	14.20	13.60	12.30	0.20	4.80	8.90	9.80	9.10	7.80	11.60	16.50	18.50	18.50	18.10	16.80
460	69	45	77	127	109	71	17129	17525	16317	14817	14999	14201	3.70	8.20	11.30	11.90	11.70	10.20	-1.60	2.60	6.50	7.60	7.30	5.70	9.00	13.80	16.20	16.30	16.20	14.70
703	84	90	116	133	122	103	20173	20285	17991	16493	16416	15122	9.00	11.90	14.20	15.10	14.90	13.50	2.30	6.00	9.40	10.60	10.30	8.70	15.70	17.80	19.00	19.60	19.40	18.30
704	3	16	47	87	91	39	20557	21749	20734	18777	18656	17405	4.80	8.80	12.30	12.40	11.70	10.30	-3.20	1.30	5.80	6.90	6.40	4.30	12.70	16.40	18.70	18.00	17.10	16.30
705	46	72	124	142	124	113	18664	19638	17912	16980	17088	16023	8.70	11.90	15.00	16.00	15.70	13.90	2.50	6.00	9.70	10.90	10.50	8.80	14.80	17.80	20.30	21.10	20.90	19.10
706	4	19	56	99	105	49	20493	21752	20326	18437	18277	17001	5.60	9.50	13.20	13.20	12.40	10.80	-2.20	2.10	6.50	7.40	7.00	4.90	13.40	16.90	19.90	19.10	17.90	16.80
707	8	21	57	100	94	54	20269	21527	20270	18646	18478	17615	6.90	11.00	14.70	14.70	14.00	12.20	-0.40	4.00	8.10	8.70	8.20	6.40	14.20	17.90	21.20	20.70	19.80	18.10
708	7	18	50	88	81	45	19968	21112	19681	18142	18167	17153	4.70	8.80	12.50	12.50	11.90	10.30	-3.20	1.40	5.80	6.60	6.10	4.20	12.50	16.10	19.20	18.50	17.80	16.40
710	17	18	31	57	69	52	20719	21990	20520	18593	17851	17110	3.30	7.10	11.10	11.20	10.60	8.90	-4.20	-0.20	4.70	5.70	5.50	3.40	10.80	14.30	17.60	16.60	15.60	14.40

712	7	20	55	98	89	52	20188	21367	20183	18554	18406	17429	6.90	10.80	14.40	14.50	13.80	12.20	-0.70	3.60	7.80	8.60	7.90	6.00	14.40	18.00	21.10	20.50	19.70	18.40
714	7	21	55	114	132	64	20599	21900	20626	18694	18053	17153	5.50	9.50	13.30	13.20	12.40	10.90	-2.50	1.60	6.20	7.30	6.90	4.70	13.60	17.30	20.30	19.20	18.00	17.10
715	24	26	42	65	73	61	20857	22041	20511	18380	17759	17085	3.80	7.40	11.30	11.40	10.60	9.10	-4.80	-0.90	4.20	5.50	5.00	2.70	12.50	15.60	18.40	17.20	16.20	15.50

Table S4 Quality control and mapping statistics of sequencing data for the 157 naked barley accessions.

Sample	Reads	Base_Counts(bp)	Q20(reads,%)	Q30(reads,%)	MatchedReads	Percent(%)	CovBase	Genomic Coverage (%)
2	13,902,708	1,618,068,182	99.84	96.94	9,281,180	66.76	138,047,133	2.86
3	11,838,172	1,375,542,508	99.82	96.69	8,001,739	67.59	131,188,948	2.71
4	21,837,752	2,516,754,240	99.84	97	13,859,603	63.47	153,460,672	3.17
5	14,956,068	1,718,065,980	99.84	96.88	10,042,537	67.15	143,425,733	2.97
6	22,459,732	2,567,727,218	99.84	97.04	14,804,044	65.91	171,424,773	3.55
7	23,411,428	2,702,555,722	99.84	96.81	15,797,839	67.48	184,989,468	3.83
8	16,141,208	1,848,886,656	99.84	97.01	10,826,927	67.08	148,681,042	3.08
9	13,528,228	1,555,271,898	99.85	97.08	9,103,871	67.3	128,727,547	2.66
10	24,792,442	2,873,520,202	99.84	96.96	16,685,497	67.3	166,112,445	3.44
11	16,782,582	1,941,068,832	99.84	96.89	10,928,288	65.12	157,469,417	3.26
12	14,965,598	1,709,984,546	99.84	96.91	10,060,633	67.23	141,927,975	2.94
13	23,370,778	2,680,376,946	99.84	97.02	15,641,456	66.93	174,065,886	3.60
14	14,593,266	1,676,378,888	99.84	96.93	9,808,229	67.21	129,436,807	2.68
15	23,990,094	2,755,959,504	99.84	97.02	16,188,676	67.48	168,633,772	3.49
16	17,779,070	2,045,254,570	99.84	96.91	11,807,164	66.41	142,731,216	2.95
17	29,213,162	3,336,698,438	99.84	97.03	19,665,161	67.32	187,753,336	3.88
19	34,792,896	4,104,743,274	99.82	95.9	23,016,420	66.15	185,300,960	3.83
21	19,513,124	2,256,327,516	99.84	96.86	13,186,453	67.58	158,753,402	3.28
23	18,958,362	2,196,983,543	99.81	96.58	12,750,640	67.26	161,416,817	3.34
24	29,603,170	3,501,894,266	99.82	95.77	19,659,641	66.41	179,584,486	3.72
25	35,014,522	4,153,735,838	99.81	95.58	23,230,922	66.35	186,403,557	3.86
27	23,020,808	2,671,630,520	99.84	96.91	15,241,181	66.21	165,381,813	3.42
28	25,540,068	2,953,575,854	99.83	96.78	17,329,434	67.85	170,863,078	3.53
30	30,759,638	3,542,356,833	99.84	97	20,495,968	66.63	180,236,047	3.73

31	12,253,082	1,423,144,263	99.82	96.88	8,590,676	70.11	111,714,383	2.31
32	11,885,166	1,354,167,557	99.71	94.25	7,643,043	64.31	127,727,136	2.64
33	25,074,222	2,912,820,295	99.83	96.88	16,832,029	67.13	181,724,498	3.76
34	12,454,826	1,416,431,819	99.68	93.84	8,067,112	64.77	132,206,202	2.74
36	18,515,568	2,122,598,660	99.84	97.03	11,731,108	63.36	149,010,799	3.08
37	23,865,932	2,755,525,748	99.84	97.08	15,286,499	64.05	169,167,435	3.50
38	24,509,720	2,820,591,330	99.85	97.07	16,540,341	67.48	186,844,300	3.87
39	26,021,136	2,982,303,052	99.81	96.74	17,473,543	67.15	175,637,124	3.63
40	29,224,514	3,349,797,084	99.84	97.09	16,692,002	57.12	170,270,952	3.52
42	36,425,270	4,172,037,362	99.86	97.3	24,535,902	67.36	194,060,149	4.01
43	34,724,794	4,011,794,608	99.85	97.14	22,798,350	65.65	190,697,232	3.95
44	25,621,320	2,955,611,912	99.84	97.05	17,183,472	67.07	171,512,861	3.55
46	11,793,576	1,332,369,734	99.69	94.07	7,586,462	64.33	127,423,980	2.64
47	16,254,556	1,887,978,264	99.84	96.89	10,869,414	66.87	146,889,131	3.04
102	15,154,424	1,738,539,056	99.69	94.05	10,002,492	66	146,058,711	3.02
104	20,902,984	2,369,375,792	99.65	93.77	13,678,872	65.44	155,354,657	3.21
109	20,369,576	2,318,086,164	99.76	96.12	13,593,105	66.73	150,397,873	3.11
111	35,601,542	4,087,897,342	99.73	95.77	23,668,982	66.48	186,188,331	3.85
112	33,535,624	3,885,422,272	99.73	95.75	22,713,416	67.73	188,948,080	3.91
113	36,850,764	4,197,730,072	99.73	95.84	24,567,370	66.67	186,046,465	3.85
116	26,201,660	2,953,006,418	99.76	96.16	17,555,307	67	183,340,916	3.79
117	20,907,398	2,372,063,840	99.74	95.89	13,879,331	66.38	147,276,952	3.05
118	24,808,426	2,828,115,586	99.7	94.31	16,245,772	65.48	172,696,613	3.57
119	13,747,498	1,572,581,694	99.69	93.96	9,093,381	66.15	135,512,816	2.80
120	38,211,248	4,347,076,398	99.75	95.88	24,850,669	65.03	202,727,665	4.19
121	27,322,170	3,107,170,154	99.76	95.97	17,975,663	65.79	173,715,210	3.59

123	48,958,228	5,575,137,740	99.75	95.95	32,243,179	65.86	217,506,444	4.50
125	18,556,072	2,121,317,566	99.69	94.09	12,288,721	66.22	153,698,785	3.18
127	27,609,128	3,130,278,940	99.75	95.95	15,445,655	55.94	165,461,312	3.42
128	11,785,382	1,323,900,031	99.66	93.84	7,620,880	64.66	132,374,902	2.74
129	28,157,472	3,204,689,894	99.72	95.46	17,728,874	62.96	182,371,370	3.77
133	33,213,920	3,806,344,956	99.75	95.9	21,437,550	64.54	179,016,252	3.70
134	16,245,398	1,857,389,377	99.69	94.04	10,357,430	63.76	143,660,141	2.97
135	32,822,622	3,717,161,933	99.75	95.81	21,912,276	66.76	183,112,212	3.79
136	13,281,688	1,498,378,502	99.7	94.22	8,493,583	63.95	131,882,554	2.73
137	45,069,462	5,112,362,559	99.76	96.06	29,659,910	65.81	210,799,741	4.36
139	38,051,944	4,351,947,696	99.74	95.95	25,148,459	66.09	185,173,849	3.83
140	23,324,494	2,615,397,213	99.72	94.35	12,137,132	52.04	151,463,159	3.13
141	35,267,030	4,004,593,051	99.75	95.96	23,562,838	66.81	193,499,184	4.00
142	40,695,028	4,602,200,578	99.76	96.08	26,761,166	65.76	198,384,273	4.10
143	19,233,704	2,192,268,032	99.7	94.24	12,628,991	65.66	157,454,085	3.26
144	40,957,744	4,656,472,128	99.75	96.11	26,977,601	65.87	203,690,591	4.21
145	54,327,684	6,204,523,222	99.75	96.03	36,103,493	66.46	229,742,032	4.75
146	62,268,984	7,081,780,160	99.69	95.49	41,585,914	66.78	227,938,885	4.72
147	47,769,074	5,394,455,618	99.75	96.06	30,889,000	64.66	207,515,417	4.29
148	20,424,304	2,308,327,384	99.7	94.22	12,963,789	63.47	157,920,864	3.27
149	52,438,440	5,881,612,950	99.78	96.38	32,888,347	62.72	212,624,191	4.40
171	19,001,520	2,222,152,982	99.77	96.06	12,841,299	67.58	151,125,617	3.13
172	31,859,046	3,715,824,951	99.78	96.25	21,343,706	66.99	192,995,489	3.99
175	50,295,294	5,834,464,173	99.78	96.22	33,649,546	66.9	233,250,108	4.83
176	32,772,042	3,805,444,151	99.78	96.28	21,413,418	65.34	196,531,429	4.07
177	18,795,644	2,197,846,910	99.78	96.33	12,549,816	66.77	144,492,639	2.99

178	23,939,014	2,706,133,071	99.7	94.22	15,910,067	66.46	170,246,073	3.52
180	27,676,792	3,239,140,612	99.78	96.25	18,501,265	66.85	176,404,206	3.65
182	24,433,450	2,843,372,244	99.78	96.22	14,970,244	61.27	164,331,433	3.40
184	42,877,094	4,790,850,331	99.53	92.5	25,852,006	60.29	145,924,829	3.02
185	15,853,714	1,844,601,668	99.77	96.15	10,176,918	64.19	141,803,879	2.93
186	64,356,298	7,345,552,610	99.78	96.51	18,489,506	28.73	172,048,259	3.56
199	38,395,658	4,528,946,387	99.77	96.19	25,962,905	67.62	183,763,359	3.80
200	47,739,350	5,394,015,031	99.8	96.56	18,372,688	38.49	174,835,188	3.62
201	35,800,776	4,131,073,690	99.78	96.37	20,550,248	57.4	185,590,289	3.84
203	33,647,140	3,896,078,520	99.78	96.33	21,390,845	63.57	193,597,906	4.01
204	31,345,388	3,618,769,140	99.78	96.47	18,288,338	58.34	183,082,227	3.79
205	51,495,686	6,007,341,280	99.78	96.42	33,278,350	64.62	226,376,925	4.68
206	44,192,612	5,165,658,500	99.73	95.88	28,994,314	65.61	205,059,627	4.24
207	39,371,910	4,564,717,472	99.78	96.43	25,670,121	65.2	202,257,244	4.18
208	58,608,276	6,736,085,450	99.79	96.63	28,786,817	49.12	192,999,333	3.99
209	26,856,432	3,081,679,286	99.79	96.53	15,009,380	55.89	174,226,111	3.60
212	91,721,308	10,261,217,444	99.81	96.87	13,885,872	15.14	157,389,089	3.26
214	27,447,208	3,121,340,230	99.7	94.37	17,912,899	65.26	180,930,253	3.74
215	51,871,124	6,055,434,744	99.78	96.28	34,732,344	66.96	214,938,555	4.45
217	40,119,716	4,425,068,652	99.79	96.34	13,897,397	34.64	157,622,245	3.26
218	28,003,522	3,115,023,711	99.77	96.03	13,338,730	47.63	153,541,723	3.18
220	30,628,128	3,471,814,736	99.78	96.19	20,009,036	65.33	173,864,786	3.60
221	27,441,602	3,118,814,615	99.76	95.97	18,094,057	65.94	167,536,564	3.47
224	55,227,684	6,122,581,258	99.78	96.28	17,475,827	31.64	164,732,596	3.41
229	54,673,652	6,214,699,830	99.77	95.98	36,837,139	67.38	247,373,929	5.12
230	42,875,196	4,833,357,106	99.47	91.85	27,895,709	65.06	157,812,884	3.26

277	29,640,452	3,339,735,502	99.75	95.72	19,451,923	65.63	176,453,505	3.65
279	34,413,552	3,895,035,818	99.78	96.01	22,329,065	64.88	176,931,838	3.66
280	37,253,124	4,241,188,848	99.76	95.97	25,831,064	69.34	180,105,418	3.73
281	44,038,850	5,011,692,285	99.77	96.07	29,157,854	66.21	201,254,362	4.16
282	46,426,662	5,299,549,143	99.77	96.04	30,461,245	65.61	198,066,234	4.10
362	52,783,024	5,957,244,426	99.78	96.28	34,743,534	65.82	216,512,752	4.48
366	47,606,414	5,346,717,594	99.79	96.47	28,502,235	59.87	199,364,484	4.12
367	84,767,382	9,385,434,398	99.79	96.5	41,029,206	48.4	229,844,758	4.76
370	29,994,916	3,358,942,060	99.79	96.42	19,778,571	65.94	192,782,513	3.99
371	24,370,584	2,737,848,578	99.78	96.22	16,017,286	65.72	184,230,998	3.81
372	27,076,260	3,151,429,440	99.75	96.15	14,716,388	54.35	156,868,943	3.25
374	28,613,544	3,241,651,606	99.72	94.69	18,863,160	65.92	180,015,098	3.72
376	31,217,560	3,511,487,256	99.73	94.79	20,386,896	65.31	185,823,526	3.84
379	33,888,428	3,800,645,362	99.52	92.38	22,073,039	65.13	138,097,574	2.86
380	25,815,088	3,004,174,372	99.73	95.9	16,902,626	65.48	175,965,317	3.64
381	21,603,970	2,497,635,967	99.75	96.1	14,382,365	66.57	163,109,114	3.37
382	27,685,822	3,124,034,834	99.72	94.57	17,739,466	64.07	179,460,687	3.71
383	33,630,994	3,795,709,176	99.71	94.35	21,569,518	64.14	194,038,629	4.01
385	45,299,940	5,025,401,498	99.78	96.47	9,490,749	20.95	139,874,648	2.89
386	14,944,442	1,792,287,071	99.77	94.82	9,862,789	66	138,258,997	2.86
387	12,332,046	1,480,864,743	99.75	94.5	8,336,606	67.6	130,386,289	2.70
389	15,357,366	1,837,687,037	99.75	94.75	10,214,553	66.51	140,037,800	2.90
390	11,674,996	1,392,544,462	99.75	94.62	7,785,027	66.68	123,898,236	2.56
391	27,378,208	3,151,892,956	99.76	96.17	17,492,002	63.89	181,803,540	3.76
395	63,651,812	7,333,681,572	99.75	96.12	35,394,648	55.61	229,601,457	4.75
396	28,162,540	3,155,590,380	99.78	96.45	14,101,056	50.07	162,880,524	3.37

399	18,234,690	2,162,163,979	99.77	94.91	12,002,308	65.82	154,763,087	3.20
404	19,745,098	2,297,726,239	99.75	96.19	13,169,186	66.7	150,181,537	3.11
407	44,239,828	5,192,861,608	99.73	95.92	29,383,354	66.42	199,415,189	4.13
408	23,202,292	2,699,052,062	99.75	95.99	15,188,496	65.46	174,129,910	3.60
409	33,533,152	3,750,047,218	99.77	96.34	11,930,853	35.58	151,382,096	3.13
413	39,043,440	4,496,650,808	99.75	96.14	23,483,299	60.15	199,279,394	4.12
414	27,506,602	3,262,365,435	99.77	94.75	18,582,805	67.56	188,631,734	3.90
418	19,170,748	2,271,784,402	99.77	94.88	12,795,700	66.75	157,474,767	3.26
420	18,966,640	2,183,826,826	99.75	96.01	12,195,801	64.3	140,172,414	2.90
421	12,844,668	1,529,046,256	99.78	95.03	8,484,753	66.06	125,167,402	2.59
422	40,314,334	4,686,479,090	99.75	96	27,035,124	67.06	203,274,257	4.21
426	25,429,616	3,031,636,658	99.76	94.81	16,698,648	65.67	167,630,907	3.47
428	21,763,536	2,498,779,870	99.75	96.07	12,633,873	58.05	148,486,142	3.07
438	25,332,992	2,982,316,990	99.73	95.94	17,335,208	68.43	162,347,408	3.36
439	44,011,224	4,924,787,386	99.53	92.53	28,386,076	64.5	156,477,636	3.24
455	29,119,792	3,398,560,642	99.75	96.07	19,267,966	66.17	182,514,641	3.78
456	26,155,252	3,107,246,728	99.76	94.81	17,500,355	66.91	174,167,860	3.60
458	37,464,552	4,390,109,570	99.75	96.05	25,339,083	67.63	198,295,587	4.10
460	31,053,894	3,548,594,231	99.76	96.17	19,000,359	61.19	185,273,493	3.83
703	19,864,464	2,342,739,468	99.72	94.31	13,105,537	65.97	160,911,990	3.33
704	52,960,978	5,866,994,346	99.82	96.91	26,045,151	49.18	198,721,879	4.11
705	32,294,760	3,659,168,736	99.81	96.66	21,290,954	65.93	173,079,121	3.58
706	32,239,526	3,633,334,951	99.79	96.39	20,668,453	64.11	179,360,976	3.71
707	25,712,128	2,930,839,410	99.81	96.64	16,914,475	65.78	154,051,349	3.19
708	15,673,006	1,850,448,024	99.72	94.04	10,290,213	65.66	155,610,405	3.22
710	32,230,192	3,629,114,704	99.81	96.64	21,107,372	65.49	175,594,448	3.63

712	16,783,194	1,970,981,992	99.7	94.12	11,082,619	66.03	148,359,508	3.07
714	21,790,132	2,546,365,750	99.72	94.42	14,194,519	65.14	168,157,860	3.48
715	28,752,012	3,185,315,958	99.54	92.41	17,721,781	61.64	124,731,788	2.58
Average	30,304,730	3,468,322,831	99.76	95.81	18,544,144	63.08	171,748,114	3.55

Table S5 Variation statistics of 3,123 SNPs for population structure analysis and pRDA.

	Count	percent
**upstream**	502	16.07%
**UTR5**	147	4.71%
**Exonic**	707	22.64%
**non_coding_transcript_exon_variant**	4	
**start_loss**	2	
**stop_loss**	1	
**stop_gained**	8	
**stop_retained_variant**	1	
**synonymous_variant**	351	
**missense_variant**	340	
**Intronic**	79	2.53%
**Splicing**	3	0.10%
**UTR3**	126	4.03%
**Downstream**	315	10.09%
**Intergenic**	1,209	38.71%
**splice_donor_variant**	1	0.03%
**splice_acceptor_variant**	2	0.06%
**splice_region_variant**	32	1.02%

Table S6 Variation statistics of 37,636 SNPs for outlier analysis.

	Count	percent
**upstream**	4,428	11.77%
**UTR5**	1,428	3.79%
**Exonic**	6,543	17.38%
**non_coding_transcript_exon_variant**	132	
**start_loss**	8	
**stop_loss**	7	
**stop_gained**	55	
**stop_retained_variant**	6	
**synonymous_variant**	3,197	
**missense_variant**	3,138	
**Intronic**	1,065	2.83%
**Splicing**	44	0.12%
**UTR3**	1,351	3.59%
**Downstream**	3,674	9.76%
**Intergenic**	18,808	49.97%
**splice_donor_variant**	27	0.07%
**splice_acceptor_variant**	29	0.08%
**splice_region_variant**	239	0.64%

Table S7 *F*st between four genetic groups.

	group1	group2	group3
group2	0.186		
group3	0.154	0.215	
group4	0.140	0.178	0.144

Table S8 Results of AMOVA for four genetic groups.

Source of variation	d.f.	Sum of squares	Variance components	Percentage of variation
Among population	3	11945.512	55.20601 Va	17.19
Within population	286	76047.813	265.90144 Vb	82.81
Total	289	87993.324	321.10746	

Table S9 The eigenvectors of RDA forward selection. The first two axes and the contribution to the total variation was presented.

Variables	F1	F2
longitude	0.5858	-0.0771
latitude	0.7378	0.0261
PC1	0.9986	0.0338
PC2	-0.0395	0.9945
PC3	0.0031	0.1003
srad6	0.4521	0.0246
prec4	-0.0732	-0.1424
srad5	0.1053	0.0484
srad4	-0.1384	0.0564
tmax6	0.0533	-0.2507
srad7	0.5754	0.0265

Table S10 The average of six nonredundant bioclimatic variables for four genetic groups.

	prec4	srad4	srad5	srad6	srad7	tmax6
group1	22.6667	18636.7708	20018.8333	19035.8125	17895.5417	18.6312
group2	27.125	19263.3125	20577.375	19379.875	17972.8125	21.0938
group3	6.6747	761.7027	815.2114	1053.9809	949.952	3.0022
group4	8.5833	763.3889	852.6875	951.25	896.5972	2.7083

Table S11 The effects of climate, geography, and genetic structure of 147 naked barley landraces calculated using pRDA. The proportion of interpretable variance represents the total constrained variance explained by the complete model; Inertia refers to variance.

pRDA model	Inertia	R2	adj R2	p(>F)	Proportion of explainable variance	Proportion of total variance
Model 1: Full model: G ~ clim. + geog. + struct.	516.7107	0.244195	0.1816848	0.001 ***	1	0.2442
Model 2: Pure structure: G ~ struct.|(clim. + geog.)	235.4605	0.1112775	0.09977219	0.001 ***	0.4558	0.1113
Model 3: Pure geography: G ~ geog.|(clim. + struct.)	35.41	0.01673582	0.005728358	0.001 ***	0.0686	0.01674
Model 4: Pure climate: G ~ clim.|(geog. + struct)	112.3	0.0530662	0.0196521	0.001 ***	0.2173	0.05307
Confounded climate/structure/geography	133.5402				0.2583	0.0631
Total unexplained	1599.2649					0.7558
Total inertia	2115.9757					1

Table S12 The eigenvectors of pure neutral model. The first two axes and the contribution to the total variation was presented.

Variables	F1	F2
srad6	0.2933	0.4995
prec4	0.2443	-0.5684
srad5	0.0165	0.3901
srad4	-0.034	0.1259
tmax6	0.3241	-0.1685
srad7	0.3178	0.5644

Table S13 A summary of putative adaptive loci associated with bioclimatic variables identified using pRDA, LFMM and EnvGWAS.

Chr.	Pos.(bp)	Method	*p*value	–log10(*p*)	*q*value	bioclimate	Annotation
chr5H	492,394,013	pRDA	2.78E-05	4.56	0.4474		5_prime_UTR_variant
chr1H	415,467,855	LFMM	1.85E-05	4.73	0.0465	prec9	downstream_gene_variant
chr1H	415,517,166	LFMM	1.62E-05	4.79	0.0455	prec9	intergenic_region
chr1H	415,529,169	LFMM	1.04E-05	4.98	0.0436	prec9	intergenic_region
chr1H	415,550,294	LFMM	2.21E-05	4.66	0.0465	prec9	intergenic_region
chr1H	417,110,925	LFMM	4.40E-06	5.36	0.0276	prec9	intergenic_region
chr1H	418,576,415	LFMM	1.60E-05	4.80	0.0455	prec9	5_prime_UTR_variant
chr1H	418,616,216	LFMM	9.57E-06	5.02	0.0436	prec9	intron_variant
chr1H	418,619,862	LFMM	5.33E-07	6.27	0.0067	prec9	synonymous_variant
chr1H	497,024,530	LFMM	9.67E-08	7.01	0.0018	prec9	missense_variant
chr1H	523,730,536	LFMM	6.65E-06	5.18	0.0358	prec9	downstream_gene_variant
chr2H	198,498,136	LFMM	3.45E-05	4.46	0.0466	prec4, prec5	intergenic_region
chr2H	276,118,573	LFMM	8.60E-07	6.07	0.0058	prec5	intergenic_region
chr2H	328,251,468	LFMM	3.56E-05	4.45	0.0466	prec4	intergenic_region
chr2H	333,974,655	LFMM	5.36E-06	5.27	0.0224	prec5	intergenic_region
chr2H	333,974,674	LFMM	2.62E-05	4.58	0.0493	prec5	intergenic_region
chr2H	438,622,045	LFMM	2.11E-05	4.68	0.0465	prec9	synonymous_variant
chr2H	464,278,576	LFMM	1.83E-06	5.74	0.0145	prec4	intergenic_region
chr2H	589,504,063	LFMM	9.29E-07	6.03	0.0058	prec5	5_prime_UTR_premature_start_codon_gain_variant
chr2H	630,451,224	LFMM	4.34E-06	5.36	0.0204	prec5	upstream_gene_variant
chr2H	719,856,837	LFMM	2.90E-05	4.54	0.0437	prec4	intergenic_region
chr2H	720,353,705*	LFMM	1.53E-05	4.82	0.0360	prec5	upstream_gene_variant
chr2H	730,687,227	LFMM	4.58E-05	4.34	0.0493	prec5	5_prime_UTR_variant
chr2H	761,134,248	LFMM	3.16E-05	4.50	0.0493	prec5	intergenic_region

chr3H	21,672,163	LFMM	3.67E-05	4.43	0.0493	prec5	intergenic_region
chr3H	34,958,420	LFMM	1.47E-06	5.83	0.0139	prec9	5_prime_UTR_variant
chr3H	35,551,500	LFMM	1.92E-06	5.72	0.0145	prec4	intergenic_region
chr3H	35,594,865	LFMM	4.30E-06	5.37	0.0147	prec4	intergenic_region
chr3H	108,807,387	LFMM	3.22E-05	4.49	0.0493	prec5	intergenic_region
chr3H	164,409,958	LFMM	1.95E-05	4.71	0.0395	prec5	intergenic_region
chr3H	183,698,406	LFMM	2.78E-06	5.56	0.0347	tavg9	upstream_gene_variant
chr3H	183,999,899	LFMM	4.51E-07	6.35	0.0169	tavg9, tmax9	upstream_gene_variant
chr3H	358,913,935	LFMM	3.79E-05	4.42	0.0493	prec5	intergenic_region
chr3H	525,399,692	LFMM	2.30E-05	4.64	0.0465	prec9	upstream_gene_variant
chr3H	584,095,427	LFMM	4.09E-06	5.39	0.0204	prec5	intergenic_region
chr3H	584,095,433	LFMM	8.86E-06	5.05	0.0256	prec5	intergenic_region
chr3H	584,358,267	LFMM	5.26E-05	4.28	0.0493	prec5	missense_variant
chr3H	617,523,738	LFMM	5.01E-05	4.30	0.0493	prec5	3_prime_UTR_variant
chr3H	621,786,474	LFMM	7.95E-06	5.10	0.0249	prec5	downstream_gene_variant
chr3H	621,786,700	LFMM	2.96E-05	4.53	0.0493	prec5	downstream_gene_variant
chr3H	637,884,849	LFMM	5.51E-05	4.26	0.0493	prec5	synonymous_variant
chr3H	637,884,868	LFMM	5.51E-05	4.26	0.0493	prec5	intron_variant
chr3H	637,884,882	LFMM	5.51E-05	4.26	0.0493	prec5	intron_variant
chr3H	637,884,914	LFMM	5.51E-05	4.26	0.0493	prec5	intron_variant
chr4H	25,273,032	LFMM	4.38E-05	4.36	0.0493	prec5	upstream_gene_variant
chr4H	25,542,171	LFMM	9.61E-08	7.02	0.0018	prec5	synonymous_variant
chr4H	479,225,288	LFMM	4.87E-05	4.31	0.0493	prec5	intergenic_region
chr4H	479,225,358	LFMM	4.87E-05	4.31	0.0493	prec5	intergenic_region
chr4H	479,225,391	LFMM	4.87E-05	4.31	0.0493	prec5	intergenic_region
chr4H	628,314,429[*]	LFMM	4.18E-06	5.38	0.0147	prec4	missense_variant

chr4H	628,324,844	LFMM	4.04E-06	5.39	0.0147	prec4	intergenic_region
chr4H	628,493,826	LFMM	4.18E-06	5.38	0.0147	prec4	missense_variant
chr5H	152,150,288	LFMM	1.99E-05	4.70	0.0395	prec5	intergenic_region
chr5H	487,359,478	LFMM	3.89E-07	6.41	0.0037	prec5	intergenic_region
chr5H	487,359,494	LFMM	3.89E-07	6.41	0.0037	prec5	intergenic_region
chr5H	490,095,740	LFMM	1.71E-06	5.77	0.0319	tavg9	intergenic_region
chr5H	567,627,515	LFMM	4.53E-05	4.34	0.0493	prec5	5_prime_UTR_variant
chr5H	600,493,261	LFMM	2.32E-08	7.63	0.0009	prec4, prec5	downstream_gene_variant
chr5H	600,578,658	LFMM	5.40E-05	4.27	0.0493	prec5	5_prime_UTR_variant
chr5H	603,520,609	LFMM	3.85E-06	5.41	0.0147	prec4	intergenic_region
chr6H	149,070,667	LFMM	3.18E-06	5.50	0.0147	prec4, prec9	intergenic_region
chr6H	151,598,697	LFMM	1.42E-05	4.85	0.0455	prec9	splice_region_variant&intron_variant
chr6H	153,983,539	LFMM	1.69E-05	4.77	0.0436	prec4, prec9	intergenic_region
chr6H	155,811,868	LFMM	2.39E-05	4.62	0.0436	prec4	intergenic_region
chr6H	160,861,004	LFMM	3.94E-05	4.40	0.0479	prec4	intergenic_region
chr6H	163,565,361	LFMM	2.78E-05	4.56	0.0436	prec4	downstream_gene_variant
chr6H	165,675,537	LFMM	6.37E-06	5.20	0.0240	prec5	upstream_gene_variant
chr6H	405,131,083	LFMM	1.79E-08	7.75	0.0007	prec9	intergenic_region
chr6H	411,488,468	LFMM	7.89E-06	5.10	0.0249	prec5	missense_variant
chr6H	545,384,949	LFMM	1.33E-05	4.88	0.0358	prec5	synonymous_variant
chr6H	545,391,626	LFMM	3.12E-05	4.51	0.0493	prec5	intron_variant
chr6H	546,749,746	LFMM	1.46E-05	4.84	0.0360	prec5	upstream_gene_variant
chr7H	204,121,205	LFMM	1.73E-06	5.76	0.0145	prec4	intergenic_region
chr7H	207,583,618	LFMM	3.71E-05	4.43	0.0466	prec4	intergenic_region
chr7H	209,644,820	LFMM	3.71E-05	4.43	0.0466	prec4	upstream_gene_variant
chr7H	210,531,561	LFMM	2.67E-05	4.57	0.0436	prec4	intergenic_region

chr7H	210,634,030	LFMM	1.97E-05	4.71	0.0436	prec4	intergenic_region
chr7H	211,426,629	LFMM	2.39E-05	4.62	0.0436	prec4	intergenic_region
chr7H	211,921,599	LFMM	1.20E-06	5.92	0.0145	prec4	intergenic_region
chr7H	211,928,316	LFMM	4.47E-07	6.35	0.0145	prec4	intergenic_region
chr7H	212,462,169	LFMM	2.67E-05	4.57	0.0436	prec4	intergenic_region
chr7H	212,462,184	LFMM	2.67E-05	4.57	0.0436	prec4	intergenic_region
chr7H	212,644,933	LFMM	2.35E-05	4.63	0.0436	prec4	intergenic_region
chr7H	213,854,546	LFMM	1.41E-05	4.85	0.0379	prec4	downstream_gene_variant
chr7H	213,936,287	LFMM	4.95E-06	5.31	0.0155	prec4	intergenic_region
chr7H	214,931,089	LFMM	2.78E-05	4.56	0.0436	prec4	intergenic_region
chr7H	214,934,257	LFMM	5.58E-06	5.25	0.0162	prec4	intergenic_region
chr7H	624,196,421	LFMM	4.17E-05	4.38	0.0490	prec4	splice_region_variant&intron_variant
chr7H	556,863,014	LFMM	4.19E-05	4.38	0.0493	prec5	intergenic_region
chr7H	573,684,269	LFMM	1.74E-05	4.76	0.0385	prec5	intergenic_region
chr7H	576,826,125	LFMM	4.78E-05	4.32	0.0493	prec5	intergenic_region
chr7H	38,883,869	LFMM	1.18E-05	4.93	0.0465	prec9	intergenic_region
chr7H	152,095,529	LFMM	2.35E-05	4.63	0.0446	prec9	downstream_gene_variant
chr1H	19,269,139	EnvGWAS	1.20E-06	5.92	0.0086	srad9	intergenic_region
chr1H	434,876,839	EnvGWAS	3.11E-12	11.51	0.0000	prec9	upstream_gene_variant
chr1H	504,106,508	EnvGWAS	3.32E-07	6.48	0.0060	srad7	intergenic_region
chr1H	538,113,269	EnvGWAS	1.49E-13	12.83	0.0000	srad4	missense_variant
chr2H	27,015,766	EnvGWAS	8.95E-10	9.05	0.0000	prec5, prec9	upstream_gene_variant
chr2H	397,522,203	EnvGWAS	4.88E-08	7.31	0.0004	prec7	intergenic_region
chr2H	584,203,708	EnvGWAS	9.71E-08	7.01	0.0012	prec5	intergenic_region
chr2H	716,449,163	EnvGWAS	6.26E-07	6.20	0.0045	prec8	upstream_gene_variant
chr2H	720,353,705*	EnvGWAS	9.60E-10	9.02	0.0000	prec5	upstream_gene_variant

chr2H	722,466,086	EnvGWAS	4.12E-11	10.39	0.0000	srad4	synonymous_variant
chr2H	727,323,942	EnvGWAS	4.26E-07	6.37	0.0040	srad8	missense_variant
chr2H	753,231,701	EnvGWAS	1.08E-06	5.97	0.0058	srad8	non_coding_transcript_exon_variant
chr2H	758,370,229	EnvGWAS	4.61E-10	9.34	0.0000	tmax5	upstream_gene_variant
chr2H	760,092,310	EnvGWAS	1.33E-13	12.88	0.0000	tmax5	intergenic_region
chr2H	765,627,976	EnvGWAS	9.37E-08	7.03	0.0018	srad8	5_prime_UTR_variant
chr3H	606,009,747	EnvGWAS	9.26E-09	8.03	0.0001	srad4	intergenic_region
chr3H	634,186,295	EnvGWAS	5.74E-08	7.24	0.0007	prec8	upstream_gene_variant
chr3H	634,581,912	EnvGWAS	1.02E-07	6.99	0.0006	prec7	upstream_gene_variant
chr3H	686,746,623	EnvGWAS	1.44E-07	6.84	0.0027	tmax4	synonymous_variant
chr4H	301,071,076	EnvGWAS	7.03E-07	6.15	0.0038	prec5	intergenic_region
chr4H	628,314,429*	EnvGWAS	8.69E-16	15.06	0.0000	srad4, srad9	missense_variant
chr5H	7,921,276	EnvGWAS	7.69E-07	6.11	0.0046	prec7, prec8	intergenic_region
chr5H	120,417,691	EnvGWAS	5.73E-08	7.24	0.0003	srad4	intergenic_region
chr5H	452,485,038	EnvGWAS	4.66E-13	12.33	0.0000	prec6, prec7, prec9, srad7, srad8	missense_variant
chr5H	485,023,709	EnvGWAS	5.25E-07	6.28	0.0040	srad8	missense_variant
chr5H	643,026,428	EnvGWAS	2.34E-08	7.63	0.0002	tmax5	intergenic_region
chr5H	650,367,670	EnvGWAS	6.73E-13	12.17	0.0000	tmax4, tmax5	intergenic_region
chr6H	6,353,354	EnvGWAS	2.84E-08	7.55	0.0004	prec7	synonymous_variant
chr6H	13,041,940	EnvGWAS	2.93E-07	6.53	0.0022	prec5	upstream_gene_variant
chr6H	274,638,437	EnvGWAS	2.86E-07	6.54	0.0022	prec5	intergenic_region
chr6H	274,638,495	EnvGWAS	4.08E-07	6.39	0.0043	srad9	intergenic_region
chr6H	559,603,685	EnvGWAS	5.19E-08	7.28	0.0003	srad4	intergenic_region
chr6H	575,948,885	EnvGWAS	1.40E-14	13.85	0.0000	prec7, prec8	upstream_gene_variant
chr6H	575,948,902	EnvGWAS	2.05E-11	10.69	0.0000	prec5, prec6, prec9	upstream_gene_variant

chr7H	10,609,308	EnvGWAS	1.40E-07	6.86	0.0026	srad9	3_prime_UTR_variant
chr7H	20,075,919	EnvGWAS	1.03E-08	7.99	0.0001	srad4	5_prime_UTR_variant
chr7H	60,223,187	EnvGWAS	1.45E-07	6.84	0.0011	tmax5	missense_variant
chr7H	70,529,503	EnvGWAS	9.84E-07	6.01	0.0058	srad8	upstream_gene_variant
chr7H	79,131,708	EnvGWAS	1.45E-07	6.84	0.0018	srad8	synonymous_variant
chr7H	161,519,775	EnvGWAS	1.27E-06	5.90	0.0068	tmax5	intergenic_region
chr7H	285,445,282	EnvGWAS	1.95E-09	8.71	0.0000	prec8	intergenic_region
chr7H	517,568,661	EnvGWAS	1.34E-13	12.87	0.0000	prec7, prec8	upstream_gene_variant
chr7H	578,374,243	EnvGWAS	3.08E-07	6.51	0.0039	prec6	upstream_gene_variant
chr7H	602,305,225	EnvGWAS	4.53E-07	6.34	0.0043	srad9	intergenic_region
chr7H	623,414,235	EnvGWAS	8.31E-07	6.08	0.0052	tmax5	stop_gained

Note: SNPs with * represented common markers identified by EnvGWAS and LFMM.

Table S14 Variation statistics for the signatures identified by pRDA, LFMM and EnvGWAS.

	pRDA	LFMM	EnvGWAS	All
**upstream**		9 (9.78%)	13 (28.89%)	21 (15.44%)
**UTR5**	1 (100%)	6 (6.52%)	2 (4.44%)	9 (6.62%)
**Exonic**		10 (10.87%)	12 (26.67%)	21 (15.44%)
**non_coding_transcript_exon_variant**			1 (2.22%)	1 (0.74%)
**stop_gained**			1 (2.22%)	1 (0.74%)
**synonymous_variant**		5 (5.43%)	4 (8.89%)	9 (6.62%)
**missense_variant**		5 (5.43%)	6 (13.33%)	10 (7.35%)
**Intronic**		5 (5.43%)		5 (3.68%)
**UTR3**		1 (1.09%)	1 (2.22%)	2 (1.47%)
**Downstream**		8 (8.70%)		8 (5.88%)
**Intergenic**		51 (55.43%)	17 (37.78%)	68 (50%)
**splice_region_variant**		2 (2.17%)		2 (1.47%)

Table S15 A summary of putative adaptive loci associated with bioclimatic variables identified using LFMM and EnvGWAS.

	chr1H	chr2H	chr3H	chr4H	chr5H	chr6H	chr7H	summary
prec4	0	4	2	3	2	5	16	32
prec5	0	11	14	6	6	8	3	48
prec6	0	0	0	0	1	1	1	3
prec7	0	1	1	0	2	2	1	7
prec8	0	1	1	0	1	1	2	6
prec9	11	1	3	0	1	5	2	23
srad4	1	1	1	1	1	1	1	7
srad7	1	0	0	0	1	0	0	2
srad8	0	3	0	0	2	0	2	7
srad9	1	0	0	1	0	1	2	5
tavg9	0	0	2	0	1	0	0	3
tmax4	0	0	1	0	1	0	0	2
tmax5	0	2	0	0	2	0	3	7
tmax9	0	0	1	0	0	0	0	1
summary	14	23	24	9	14	19	32	135

Supplementary Figures

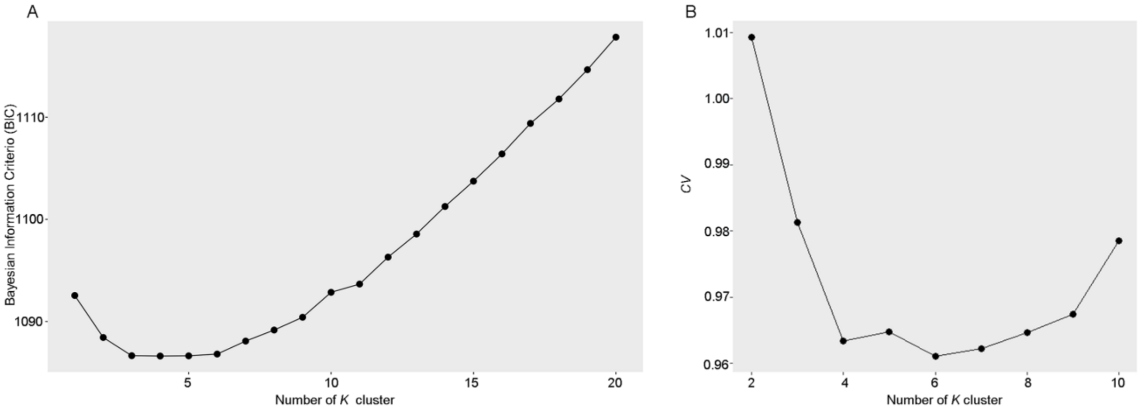

Figure S1 A Predictive accuracy of the Discriminant Analysis of Principal Components (DAPC) procedure: successive Kmeans for a 20 k levels and the computed Bayesian Information Criterion (BIC) of the corresponding models were plotted. **B** The trend of *CV* error values estimated by using Admixture with K=2-10.

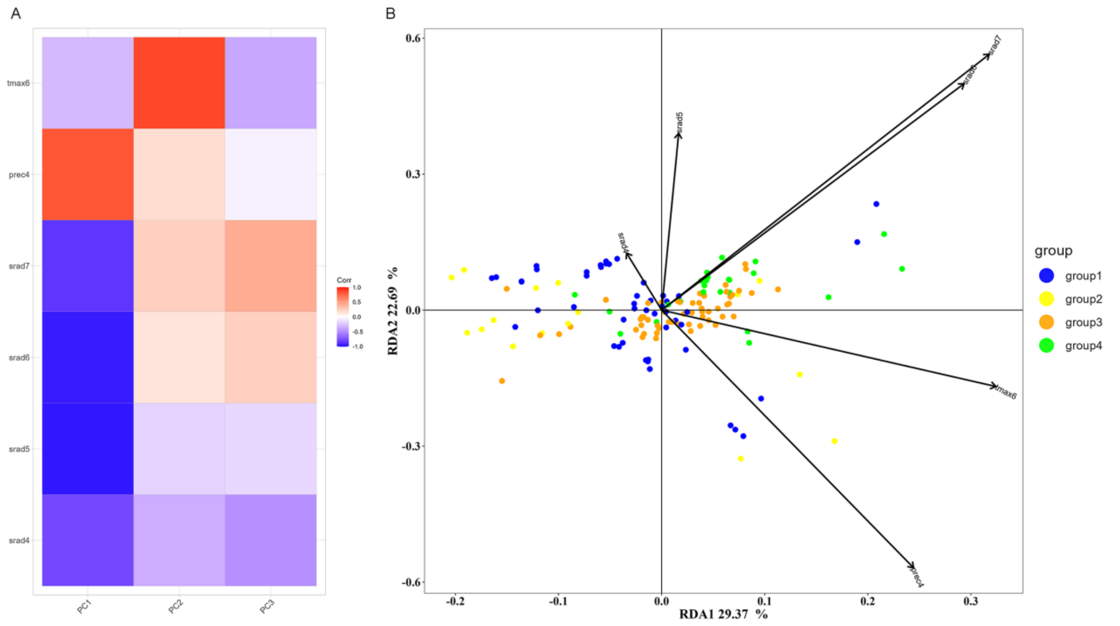

Figure S2 A Correlation between climatic variables and derived PCs, colored according to legend. **B** Pure neutral model (control on genetic structure). The biplot depicted the eigenvalues and length of eigenvectors for the RDA. Blue, yellow, orange and green dots correspond to group 1, group 2, group 3 and group 4, respectively.

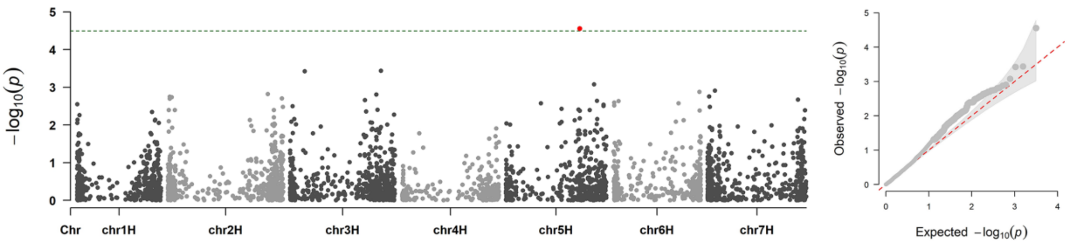

Figure S3 The signatures identified by using pRDA on the linkage disequilibrium-pruned SNP markers (n = 3,123). The horizontal dashed dark green line indicated a Bonferroni thresholds based on α =0 .05 (−log10 (*p*) = 4.495), the red dot indicated the significant locus.

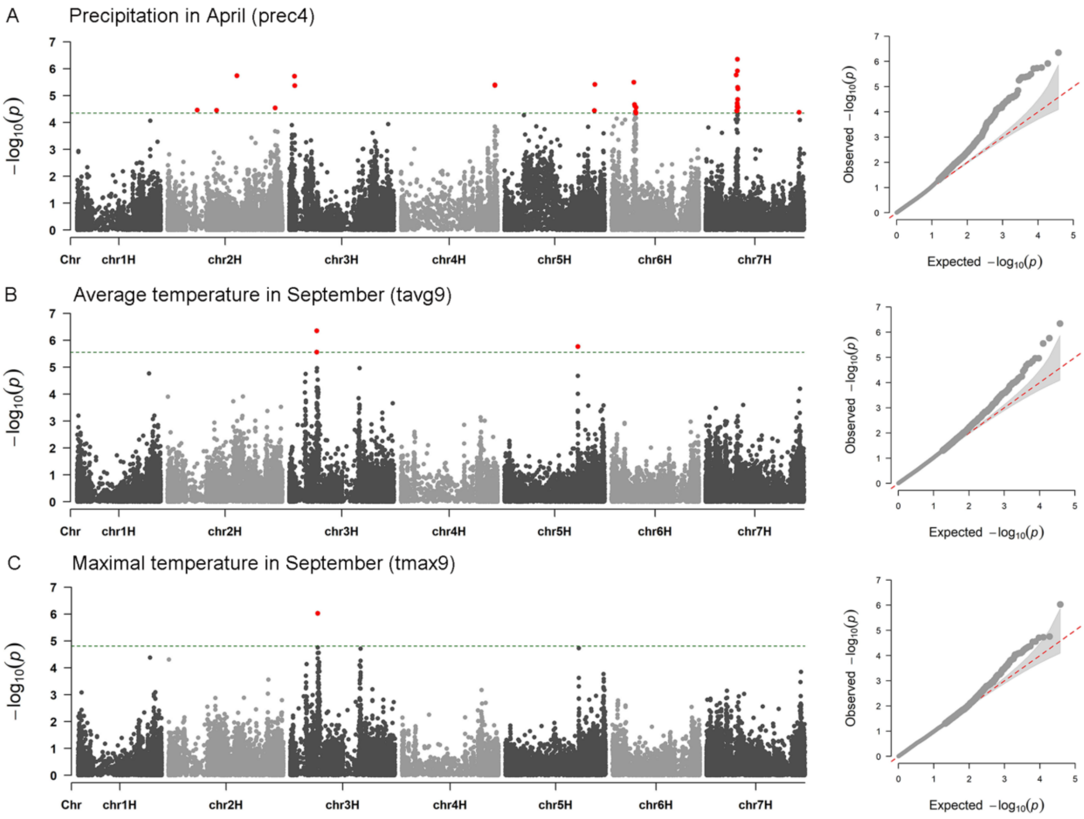

Figure S4 The candidate SNPs identified by using LFMM on the 37,636 SNP markers with MAF ≥ 0.05 (n = 37,636) for: **A** precipitation in April; **B** Average temperature in September; **C** Maximal temperature in September. The dark green line was based on false discovery rate with q-value >0.05, specific for each trait. The red dots indicated the significant loci.

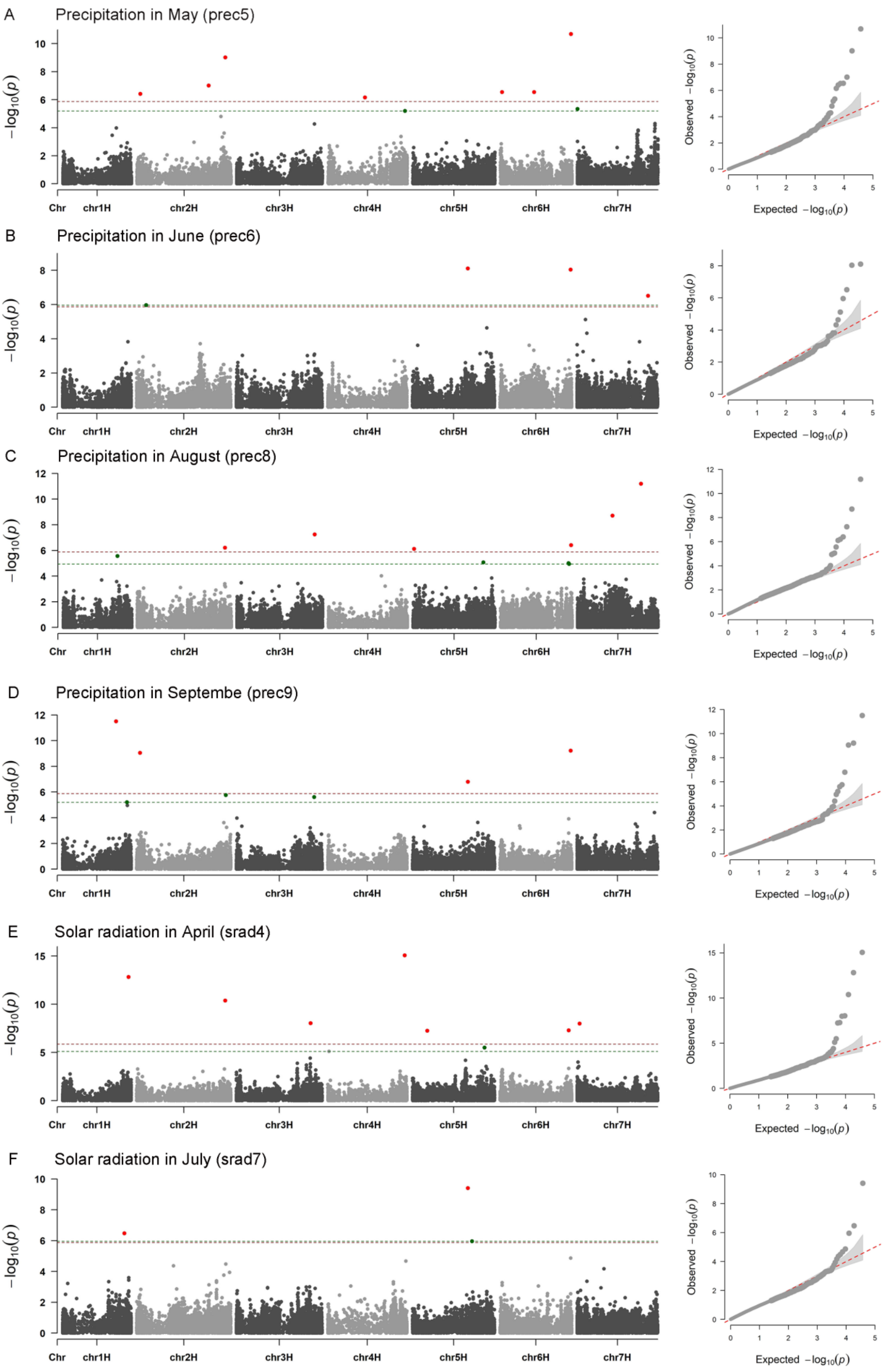
A
Precipitation in May (prec5)
B
Precipitation in June (prec6)
C
Precipitation in August (prec8)
D
Precipitation in Septembe (prec9)
E
Solar radiation in April (srad4)
F
Solar radiation in July (srad7)
$-\log_{10}(p)$
Chr
chr1H
chr2H
chr3H
chr4H
chr5H
chr6H
chr7H
Observed $-\log_{10}(p)$
Expected $-\log_{10}(p)$

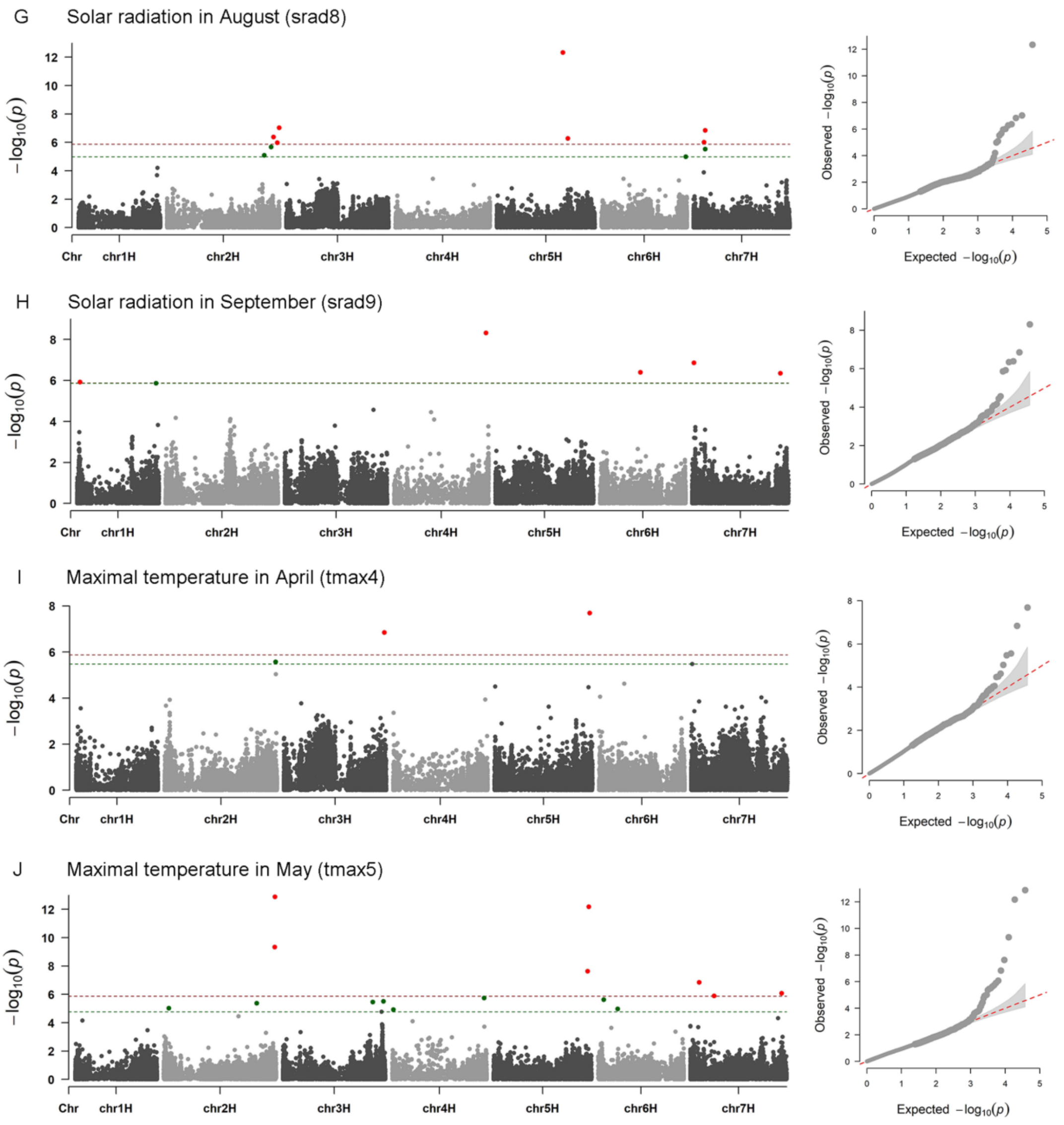

Figure S5 The signatures identified by using EnvGWAS on the 37,636 SNPs with MAF ≥ 0.05 (n = 37,636) for: **A-D** precipitation in May to September (expect for July); **E-H** solar radiation in April and solar radiation in July to September; **I-J** maximal temperature in April and May. The dark red line refers to Bonferroni correction based on α = 0.05 (− log10 (p) = 5.877) while the green line was based on false discovery rate with q-value > 0.05, specific for each trait. The red dots indicated the significant loci.

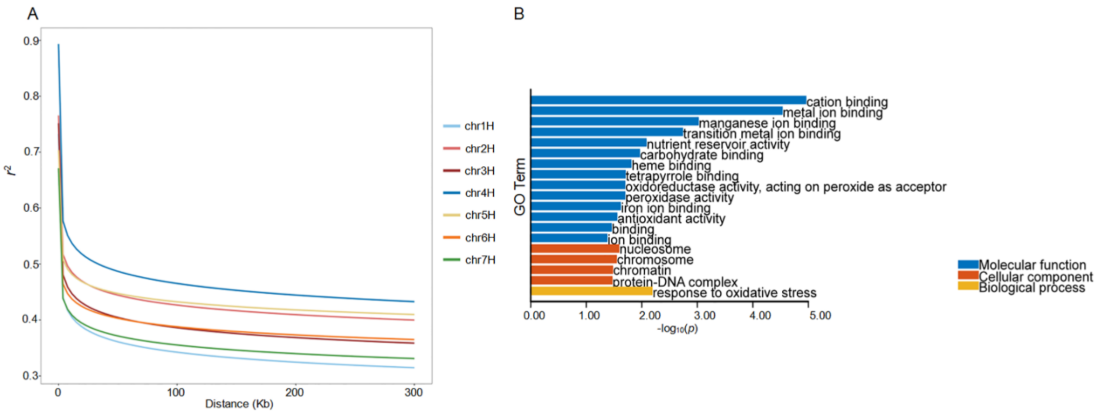

Figure S6 A Linkage disequilibrium (LD) decay. **B** The candidate genes ontology.
